# ATP2, The essential P4-ATPase of malaria parasites, catalyzes lipid-stimulated ATP hydrolysis in complex with a Cdc50 β-subunit

**DOI:** 10.1080/22221751.2020.1870413

**Published:** 2021-01-17

**Authors:** Anaïs Lamy, Ewerton Macarini-Bruzaferro, Thibaud Dieudonné, Alex Perálvarez-Marín, Guillaume Lenoir, Cédric Montigny, Marc le Maire, José Luis Vázquez-Ibar

**Affiliations:** a Université Paris-Saclay, CEA, CNRS, Institute for Integrative Biology of the Cell (I2BC), Gif-sur-Yvette, France; b Department of Medical Biochemistry and Biophysics, Umeå University, Umeå, Sweden; c Department of Clinical Medicine (FMUSP), University of São Paulo, São Paulo, Brazil; d DANDRITE, Nordic EMBL Partnership for Molecular Medicine, Department of Molecular Biology and Genetics, Aarhus University, Aarhus, Denmark; e Biophysics Unit, Department of Biochemistry and Molecular Biology, School of Medicine, Universitat Autònoma de Barcelona, Cerdanyola del Vallés, Spain

**Keywords:** Malaria, P4-ATPases, lipid flippase, PfATP2, membrane transport proteins, heterologous expression

## Abstract

Gene targeting approaches have demonstrated the essential role for the malaria parasite of membrane transport proteins involved in lipid transport and in the maintenance of membrane lipid asymmetry, representing emerging oportunites for therapeutical intervention. This is the case of ATP2, a *Plasmodium*-encoded 4 P-type ATPase (P4-ATPase or lipid flippase), whose activity is completely irreplaceable during the asexual stages of the parasite. Moreover, a recent chemogenomic study has situated ATP2 as the possible target of two antimalarial drug candidates. In eukaryotes, P4-ATPases assure the asymmetric phospholipid distribution in membranes by translocating phospholipids from the outer to the inner leaflet. In this work, we have used a recombinantly-produced *P. chabaudi* ATP2 (PcATP2), to gain insights into the function and structural organization of this essential transporter. Our work demonstrates that PcATP2 associates with two of the three *Plasmodium-*encoded Cdc50 proteins: PcCdc50B and PcCdc50A. Purified PcATP2/PcCdc50B complex displays ATPase activity in the presence of either phosphatidylserine or phosphatidylethanolamine. In addition, this activity is upregulated by phosphatidylinositol 4-phosphate. Overall, our work describes the first biochemical characterization of a *Plasmodium* lipid flippase, a first step towards the understanding of the essential physiological role of this transporter and towards its validation as a potential antimalarial drug target.

## Introduction

Malaria is a global health problem affecting over 200 millions of people worldwide, and responsible in 2018 of nearly 4,05,000 deaths around the globe, mostly children[1]. Malaria is caused by parasites of the genus *Plasmodium*, a pathogen with a complex life-cycle between the transmission vector, the mosquito *Anopheles,* and a vertebrate host*.* Five different *Plasmodium* species infect humans, *P. falciparum* being the most virulent one, accounting for ∼90% of the mortality caused by this disease. Over the last 15 years, artemisinin-combined therapies (ACT) have permitted a substantial reduction of the mortality of this disease. However, *P. falciparum* strains resistant to current treatments, particularly to artemisinin, have already appeared in different regions of south Asia, representing a serious threat to eradicate malaria as no effective vaccine is yet available [2]. The fight against malaria thus requires a continuous supply of new drugs to supplement the existing therapeutic arsenal. In fact, the combination of drugs acting on different targets and/or stages is the most efficient strategy to combat malaria as it minimizes the development of new drug-resistance mechanisms [3].

Recent efforts combining genetic disruption tools and phenotypic analysis in human and rodent malaria parasites have revealed that ∼78% of *Plasmodium*-encoded membrane transport proteins (MTPs) are either essential or necessary for normal grow and development [4–6], thus confirming their therapeutic potential [7]. Among the annotated 144 MTPs, *P. falciparum* encodes 13 P-type ATPases [7], most of them refractory to gene deletion [8]. P-type ATPases are a large family of primary transporters that utilize the energy from ATP hydrolysis to pump cations (subfamilies P1, P2 and P3)[9], lipids (subfamily P4)[10], polyamines (subfamily P5B)[11], or even to discolase single transmembrane domains (P5A subfamily)[12]. In *Plasmodium*, P-type ATPases maintain ion homeostasis during its intracellular life-cycle [13]. The *P. falciparum* Na^+^/H^+^ P-type ATPase (PfATP4) has been identified as the target of the antimalarial family of drugs spiroindolones [14]. The *P. falciparum* homologue of the sarco/endoplasmic reticulum Ca^2+^ ATPase (SERCA) pump (PfATP6) was initially proposed as an artemisinin target [15], although studies using recombinant PfATP6 did not support this [16]. Recently, gene-targeting approaches have also uncovered the important role for parasite’s progression of the P4 subfamily, also known as lipid flippases [4–6]. In eukaryotes, P4-ATPases maintain the asymmetric distribution of phospholipids in membranes by translocating phospholipids from the extracellular (or luminal) leaflet to the cytoplasmic leaflet [10], key in important physiological functions as vesicle budding and protein trafficking [17]. Moreover, new roles of these transporters have emerged in recent years as membrane lipids are also implicated in cell-signalling events. In plants, the transport of lysophospholipids mediated by a P4-ATPase is involved in root development and size of stomatal apertures [18]. Also, the virulence of some pathogens and drug disponibility have been correlated with the expression and activity of P4-ATPases [19]. The genome of *P. falciparum* contains four putative P4-ATPases: PfATP2, PfATP7, PfATP8 and PfATP11 [7], well conserved in *Plasmodium* species with the exception of PfATP11 [8]. In addition, *P. falciparum* encodes two P4-ATPase-like proteins only present in the Apicomplexa phylum: GCα and GCβ, consisting of a N-terminal P4-ATPase-like domain connected to a C-terminal guanylate cyclase domain. While there is some discrepancy with regard the vital role of ATP7 and ATP8 [4,5], all the studies agree that the activites of PfATP2 and its ortholog in the mouse malaria parasite *P. berguei* are completely irreplaceable during the blood stages. Moreover, in a recent study [20], gene duplication of the gene encoding PfATP2 was associated with drug-resistant phenotypes against two drugs antimalarial compounds. This eventually situates PfATP2 either, as the target of these two antimalarial compounds or responsible of reducing the local concentration of these drugs at the target site.

Most P4-ATPases form heterodimeric complexes with members of the Cdc50/LEM3 protein family, and this association is essential for trafficking of the P4-ATPase/Cdc50 complex [21], and for activity [22]. The recent Cryo-EM structures of four P4-ATPases in complex with their respective Cdc50 subunits have revealed the structural basis of P4-ATPase/Cdc50 association [23–28] *P. falciparum* encodes three putative Cdc50 proteins (annotated as Cdc50A, Cdc50B and Cdc50C) also well conserved in *Plasmodium* species. Gene-disruption of either of these putative Cdc50 proteins affects parasite’s progression in the blod stages [6]. A recent study in *P. yoelii* has shown that Cdc50A interacts and stabilizes the GCβ/Cdc50A complex, mandatory for the gliding motility and midgut traversal of the ookinetes in the mosquito vector and, consequently, for parasite’s transmission [29]. Also, its related Cdc50A homolog in *Toxoplasma gondii* is mandatory for bringing its GCβ partner to the plasma membrane prior cellular egression [30].

ATP2 and the rest of *Plasmodium*-encoded P4-ATPases still remain as putative transporters waiting for functional annotation. In addition, and despite the importance of the P4-ATPase/Cdc50 association, to date, the identity of the Cdc50-interacting partners of ATP2 have not been revealed, neither in the parasite nor *in vitro* using recombinant proteins. Moreover, the biological role of such possible association is still unknown. Here, using recombinantly-produced proteins, we demonstrate that the *Plasmodium chabaudi* ATP2 (PcATP2) forms heterodimers with two of the three *Plasmodium*-encoded Cdc50 proteins: PcCdc50B and PcCdc50A. Moreover, PcATP2/PcCdc50B displayed lipid-stimulated ATPase activity in the presence of two putative phospholipid substrates, and this activity was slighly upregulated by the presence of PI4P. We provide the first study of the function and structural organization of a *Plasmodium* lipid flippase, an emerging antimalarial target candidate.

## Materials and methods

### Molecular cloning

Synthetic cDNAs encoding three putative ATP2 orthologs from *P. falciparum (PF3D7_1219600)*, *P. berghei* (PBANKA_1434800), and *P. chabaudi (PCAHS_1436800)*, and the three Cdc50 subunits encoded per specie: *P. falciparum* (PF3D7_0719500, PF3D7_1133300, and PF3D7_1029400), *P. berghei* (PBANKA_061700, PBANKA_091510, and PBANKA_051340), and *P. chabaudi* (PCHAS_061870, PBANKA_091510, and PBANKA_051340) were ordered to Genscript (Piscataway, NJ). All the cDNAs were codon-optimized by the provider for *S. cerevisiae* expression using the proprietary algorithm OptimumGene^TM^. In all cloning steps, we used the *E. coli* XL1-Blue strain and the Luria–Bertani medium with the appropriate antibiotic.

*Single expression vectors*: The cDNAs encoding the three ATP2 orthologs were cloned in the pYeDP60 expression vector [31] between the *EcoRI* and *NotI* restriction sites, yielding the pYeDP60-ATP2-TEV-BAD plasmid that contains in downstream position the tobacco etch virus (TEV) protease cleavage-site coding sequence followed by the biotin acceptor domain (BAD) coding sequence. To obtain the N-terminal BAD tagged ATP2 constructs, pYeDP60-BAD-TEV-ATP2, we amplified by PCR the ATP2 sequences incorporating the *PmeI* and *SacI* restrictions sites at both, the 5′ and 3′ ends, and cloned into the pYeDP60 vector. The PcATP2-D596N mutant was obtained using the QuikChange II XL site-directed mutagenesis kit (Agilent). The nine Cdc50 subunits sequences were cloned in the pYeDP60 vector between the *EcoRI* and *BamHI* restriction sites. The resulting pYeDP60-Cdc50-TEV-10xHis vectors contain the TEV site followed by a 10xHis tag at the C-terminal end of the proteins.

*Co-expression vectors*: pYedBAD-BAD-Drs2p/Cdc50p-His was cloned previously [31]. To construct the other co-expression vectors we used a previous strategy. The fragments containing the cassette Promoter-Cdc50-TEV-His10-Terminator from each pYeDP60-Cdc50-TEV-10xHis vector were amplified by PCR, incorporating the *SbfI* restriction site at both the 5′ and the 3′ ends. The PCR fragments were then cloned at the *SbfI* site of the corresponding species-specific single-expression vectors pYeDP60-ATP2-TEV-BAD and pYeDP60-BAD-TEV-ATP2.

### Introduction of the superfolder Green fluorescent protein

The cDNA encoding the superfolder Green Fluorescent Protein (GFP) was PCR amplified, introducing a *NotI* site followed by the human Rhinovirus 3C protease sequence (3C-protease) at the 5′, and the *XmaI* site at the 3′. The PCR product was then cloned into the *NotI* and *XmaI* sites of both the single-expression pYeDP60-PcATP2-TEV-BAD and the co-expression pYeDP60-PcATP2-TEV-BAD/PcCdc50B-10xHis vectors to obtain the pYeDP60-PcATP2-3C_Protease-GFP-TEV-BAD and the pYeDP60-PcATP2-3C_Protease-GFP-TEV-BAD/PcCdc50B-10xHis vectors. Using an identical cloning strategy, we also made the same constructs but without the BAD domain by adding a stop codon next to the *XmaI* site, leading to the the pYeDP60-PcATP2-D596N-3C_Protease-GFP/PcCdc50B-10xHis vector. To introduce the sGFP at the C-terminal end of PcCdc50B, we amplified by PCR a DNA fragment encoding the 3C_protease followed by the GFP with *BamHI* sites at both the 5′ and the 3′ ens. The sequence was then cloned at the *BamH1* site of pYeDP60-PcCdc50B-TEV-10xHis vectors, resulting in the pYeDP60-PcCdc50B-3C_protease-GFP-10xHis vector.

### Yeast transformation

The different proteins and protein complexes were expressed in the *Saccharomyces cerevisiae* strain W3031b Gal4-2 (α, leu2, his3, trp1::TRP1-GAL10-GAL4, ura3, ade2-1, canr, cir+) [32]. For yeast transformation, 5 ml of non-transformed W3031b Gal4-2 strain was cultured in S6 AU minimal media (0.1% (w/v) Bacto^TM^ Casamino Acids, 0.7% (w/v) Yeast nitrogen base (without amino acids and with ammonium sulphate), 2% (w/v) glucose, 20 µg/mL adenine and 20 µg/mL uracil) for 24 h at 28°C. Cells were mixed with ∼1 μg of plasmid DNA and 100 μg of salmon sperm DNA (denatured at 100°C for 5 min and cooled down on ice). After vortexing, 500 μL of 40% (w/v) PEG 4000, 100 mM lithium acetate, 10 mM Tris-HCl pH 7.5 and 1 mM EDTA and 20 μL of 1 M DTT was added. The tube was vortexed again and left overnight at room temperature. The next morning the tube was centrifuged at 350 *× g* for 2 min, the supernatant was removed, and the cells were re-suspended in 100 μL of S6A minimal media (SAU without uracil) and plated on S6A-agar plates. The plates were left in the incubator at 28°C for 3–5 days until the transformed colonies appeared.

### Protein expression in *S. cerevisiae*

Transformed yeast colonies were pre-cultured in 5 mL of S6A media or 24 h at 28°C. For small-scale expression tests, the pre-culture was diluted to OD_600_ of 0.2 in 20 mL of YPGE2X rich media (2% (w/v) Bacto^TM^ Peptone, 2% (w/v), yeast extract, 1% (w/v) glucose and 2.7% (w/v) ethanol) and cultured for 30 h at 28°C. Then, the cell culture was cooled down to 18°C, and 20 g/L of galactose was added to induce protein expression for 18 h at 18°C.

For large-scale cultures used for membrane fractionation, the first pre-culture was diluted to a OD_600_ of 0.1 in 50 mL of S6A media and incubated at 28°C for another 24 h. Then, this second pre-culture was diluted to an OD_600_ of 0.05 in 500 mL of YPGE2X media. The cells were grown for 36 h at 28°C to consume the majority of glucose. The culture flasks were cooled down to 18°C, and 2% (w/v) of galactose were added to induce protein expression. After 13 h at 18°C, a second addition of 2% (w/v) galactose was done and the culture was left for five more hours. The cells were centrifuged at 4000 *× g* during 10 min at 4°C and washed two times with cold water. The cell-pellet was weighted and re-suspended in 2 mL of cold TEKS buffer (50 mM Tris-HCl pH 7.5, 1 mM EDTA, 0.1 M KCl and 0.6 M sorbitol) per gram of cells. After incubation at 4°C for 15 min, the cells were centrifuged and the resulting pellet was flash-frozen in liquid nitrogen and stored at −80°C.

### Membrane preparation

To analyze the expression of the proteins induced in the small-scale cultures, a total membrane preparation was done. A volume of each culture corresponding to 10 optical density units at 600 nm was centrifuged at 800 *×* *g* for 10 min and 4°C. Cells were re-suspended in 1 mL of ice-cold TEPI buffer (50 mM Tris-HCl pH 6.8, 5 mM EDTA, 20 mM NaN_3_) supplemented with SigmaFast^TM^ EDTA-free Protease Inhibitor Cocktail (PIC) tablets (Sigma-Aldrich) and 1 mM phenylmethylsulfonyl fluoride (PMSF, Sigma-Aldrich) and transferred into 1.5 mL Eppendorf tubes. After centrifugation at 1000 *×* *g* for 10 min and 4°C, the pellet was re-suspended in 100 µL of ice-cold TEPI buffer, and cells were broken after adding 100 µL of glass beads (0.5 mm of diameter) and vortexing for 25 min at 4°C. Then, TEPI buffer was added to a final volume of 1 mL and samples were centrifuged for 5 min at 500 *×* *g* and 4°C. The supernatant was then centrifuged at 100 000 *× g* for 90 min and 4°C. The resulting membrane pellet was re-suspended directly in 200 µL of SDS-PAGE loading buffer containing urea, heated at 30°C during 10 min, and subjected to SDS-PAGE and western blot analysis.

Frozen cell-pellets from large-scale cultures were resuspended in 1 mL of TES buffer (50 mM Tris-HCl pH 7.5, 1 mM EDTA and 0.6 M sorbitol) per gram of cell-pellet, supplemented with PIC and 1 mM PMSF. The cells were broken with 0.5 mm glass beads using the planetary mill Pulverisette 6 (Fritsch). The broken crude extract was recovered and the beads were washed with 1.5 mL of ice-cold TES buffer per gram of cell-pellet. The pH of the broken cells was adjusted to 7.5, and centrifuged at 1000 *× g* for 20 min at 4°C. The resulting supernatant (S1) was centrifuged at 20 000 *× g* for 20 min at 4°C to obtain the second pellet (P2). The resulting supernatant from the previous step (S2) was then centrifuged at 125,000 *× g* during 1 h at 4°C, obtaining the third pellet (P3). P3 pellet was re-suspended in 0.2 mL of HEPES-sucrose buffer (20 mM HEPES pH 7.6, 0,25 M sucrose) per gram of cell-pellet. Aliquots of all membrane pellets were flash-frozen in liquid nitrogen and stored at - 80°C. An appropriate amount of membranes containing the desired total protein amount was mixed with loading buffer containing urea, heated at 30°C during 10 min, and subjected to SDS-PAGE and western blot analysis.

### Protein detection by western blotting

Total protein concentration in the different samples was measured by the Bicinchoninic Acid Assay (BCA). Samples were subjected to SDS-PAGE and proteins were transferred to Polyvinylidene difluoride (PVDF) membranes (Immobilon®-P, Merck). The ATP2 proteins fused to the BAD tag were detected with the Avidin HRP-conjugate probe (Invitrogen), diluted to 1:20,000 in 5% (w/v) milk in PBS-Tween buffer (PBS + 0.02% (v/v) Tween®20). Cdc50 proteins fused to the 10xHis tag were detected using the HisProbeTM-HRP conjugate (ThermoFisher) diluted to 1:2000 in 2% (w/v) BSA in PBS-Tween buffer. GFP-tagged proteins were detected with a primary mouse antibody IgG1 K Anti-GFP (Roche) diluted to 1:1000 in 5% (w/v) milk in PBS-Tween buffer, followed by a incubation with a second Goat Anti-Mouse IgG-HRP conjugate antibody (Bio-Rad) diluted to 1: 3000 in 5% (w/v) milk in PBS-Tween buffer. The ECL Western Blotting Detection kit (GE healthcare) was used for western blot revelation, and luminescence was detected by a CDD camera.

### Deglycosylation assays of PcCdc50 proteins

P3 membranes co-expressing PcATP2/PcCdc50A, PcATP2/PcCdc50B or Drs2p/Cdc50p were diluted to 4 mg/mL in HEPES-sucrose buffer. 20 µg of total protein were added to a glycoprotein denaturing buffer (0.5% (w/v) SDS, 40 mM DTT) and protein denaturation was performed at 100°C for 10 min in a dry bath. Samples were cooled down on ice for 5 min and spun down at 15,000 *× g* for 10 sec. 10 µL of denatured samples were enzymatically digested with either, PGNase F or EndoH (New England Biolabs) using the buffers and protocols provided by the supplier. The reaction was carried out for 1 h at 37°C and results were analyzed by western blot.

### Detergent solubilization screenings

P2 and P3 membrane fractions were solubilized with eleven different detergents at 2 mg/mL of total protein concentration in the membranes, and 10 mg/mL of detergent concentration in solubilization buffer (20 mM Tris-HCl pH 7.8, 10% (v/v) glycerol, 150 mM NaCl, 1 mM PMSF and PIC). Solubilization was performed at 4°C or 20°C and at different incubation times. 1 h and 20°C were the conditions that showed the best results. After incubation, samples were ultracentrifugated for 1 h at 125,000 *× g* and 4°C, the pellet was resuspended in the same volume as the supernatant, and both, the pellet and the supernatant (detergent-solubilized sample) were subjected to SDS-PAGE and western blot to analyze the solubilization efficiency of each experimental condition. Detergents used in the screen: n-decyl-β-D-maltopyranoside (DM), n-undecyl-β-D-maltopyranoside (UDM) and octaethylene glycol monododecyl Ether (C12E8) from Calbiochem; n-dodecyl-β-D-maltopyranoside (DDM), lauryl maltose neopentyl glycol (LMNG), 3-[(3-Cholamidopropyl)dimethylammonio]-1-propanesulfonate hydrate (CHAPS), and n-dodecyl phosphocholine 12 (FosC12) from Anatrace; n-Octyl-β-D-glucopyranoside (OG), n-Octyl-β-D-thioglucopyranoside (OTG) from Sigma-Aldrich; and Laurydimethylaminoxide (LDAO), and 5-cyclohexyl-1-pentyl-β-D-maltoside (CYMAL-5) from Fluka. Solubilization experiments were also performed in the presence of the cholesterol derivative, cholesteryl hemisuccinate (CHS) (Sigma-Aldrich) at a 5:1 (w/w) detergent to CHS ratio.

### Co-immunoprecipitation assays

Co-immunoprecipitation assays were performed using agarose beads coupled to nanobodies recognizing the GFP (GFP-Trap^®^, ChromoTek), and following the protocol suggested by the provider. 500 µL of P3 membranes diluted to 2 mg/mL of total protein concentration were solubilized in solubilization buffer containing 10 mg/mL of DDM and 2 mg/mL CHS, during 1 h at 20°C. After incubation, membranes were ultra-centrifuged at 125,000 *× g* for 90 min at 4°C, and the supernatant was incubated for 1 h and 4°C with gentle rotation with 25 µL of GFP-Trap^®^ beads, previously equilibrated in washing buffer 1 (20 mM Tris-HCl pH 7.8, 150 mM NaCl, 0.2 mg/mL DDM, 0.04 mg/mL CHS, 10% (v/v) glycerol). After incubation, the flow-through was removed by centrifuging the sample at 100 *× g* for 30 s. The beads were then washed by adding 500 µL of ice-cold washing buffer 1 followed by centrifugation at 100 *× g* for 30 sec. A second wash was done with 500 µL of washing buffer 2 (20 mM Tris-HCl pH 7.8, 500 mM NaCl, 0.2 mg/mL DDM and 10% (v/v) glycerol). Proteins bound to the GFP-Trap^®^ beads were eluted by adding 50 µL of 0.2 M glycine buffer pH 2.5 followed by centrifugation at 100 *× g* for 30 sec. The low-pH of the elution was quickly neutralized by adding 5 µL of 1 M Tris-base pH 10.4. This elution step was repeated one more time and samples were analyzed by western blot.

### Fluorescence-detection size-exclusion chromatography

P3 membranes were solubilized as described in the previous section and 200 µL of the detergent-solubilized supernatant was injected in a Superose 6 10/300 GL gel-filtration column (GE healthcare) equilibrated with 20 mM Tris-HCl pH 7.8, 150 mM NaCl, 10% (v/v) Glycerol, 0.1 mg/mL DDM, 0.02 mg/mL CHS. Fluorescence-detection size-exclusion chromatography (FSEC) experiments were performed in an ÄKTA^TM^ purifier chromatography system (GE healthcare) with a fluorescence detector (FP-4025, JASCO) connected “in line” with the column. The excitation and emission wavelengths of the fluorescence detector were set, respectively, to 460 and 520 nm. FSEC chromatograms were plotted using Plot2 software and data was normalized.

### Generation of home-made GFP-Trap^®^ beads for ATPase assays

We first expressed and purified a nanobody directed against GFP (nanoGFP). The plasmid vector pOPINE GFP nanobody was a gift from Brett Collins (Addgene plasmid # 49172) [33]. 500 mL of *E. coli* BL21 cells harbouring this plasmid were cultured at 37°C in LB media supplemented with ampicillin. When the OD_600_ reached ∼0.6, the temperature was dropped to 20°C, and protein expression was induced with 1 mM isopropyl ß-D-1-thiogalactopyranoside (IPTG) (Sigma-Aldrich) for 20–24 h at 20°C. The cells were spun down, flash-frozen in liquid nitrogen, and stored at −80°C. The cell-pellet was re-suspended in 10 mL of lysis buffer (PBS pH 8, 0.5 M NaCl, 5 mM imidazole, 1 mM PMSF and 10 µg/µL of Lysozyme) and left for 1 h at 4°C with gentle rotation. Cells were broken by sonication, maintaining the cells always on ice. Cell lysate was centrifuged at 20,000 *× g* for 20 min at 4°C, and the supernatant was mixed with 1 mL of TALON® metal affinity beads (Clontech) previously equilibrated with equilibration buffer (PBS pH 8, 0.5 M NaCl and 5 mM imidazole), and incubated for 1 h at 4°C under gentle rotation. After incubation, the beads were sequentially washed with 20 column-volumes of equilibration buffer, 10 column-volumes of equilibration buffer with 20 mM imidazole, and 10 column-volumes of equilibration buffer with 30 mM imidazole. The nanoGFP was eluted with two steps of 5 column-volumes of equilibration buffer with 150 mM of imidazole, and 5 column-volumes of equilibration buffer with 300 mM imidazole. The elution fractions were analyzed by SDS-PAGE, pooled and concentrated using a Vivaspin® 20,5,000 Mw concentrator (Sartorius). The concentrated protein was dialyzed overnight at 4°C to remove the imidazole, further concentrated and stored at 4°C until use. The purified nanoGFP was then covalently bound to agarose beads. 1 ml of NHS-Activated Sepharose 4 Fast Flow beads (GE Healthcare Life Sciences) was washed with 10–15 column-volumes of 1 mM HCl, and equilibrated with PBS pH 7.5. Then, 1 mg of nanoGFP was added to the beads and left overnight at 4°C under gentle rotation, keeping a nanoGFP to beads volume ratio of approximately 0.5–1. After the overnight reaction, the remaining non-reacted sites were blocked by the addition of 10–15 column-volumes of 0.1 M Tris-HCl pH 8.5 followed by a 4 h incubation with this buffer at 4°C. Finally, the beads were subjected to 3 cycles of two sequential washes of 3 column-volumes of 0.1 M Tris-HCl, 0.5 M NaCl, pH 8.5 and 3 column-volumes of 0.1 M Acetate buffer, pH 5.0, 0.5 M NaCl. Finally, the home-made GFP-Trap^®^ beads were equilibrated in PBS pH 8.0 and stored at 4°C until use.

### Purification and immobilization of PcATP2/PcCdc50B in home-made GFP-Trap^®^ beads

P3 membranes expressing either PcATP2-GFP-BAD/PcCdc50B-His or PcATP2-D596N-GFP were diluted up to 5 mg/mL of total protein concentration in solubilization buffer (20 mM Tris-HCl pH 7.8, 150 mM NaCl, 100 mM KCl, 5 mM MgCl_2_, 20% (w/v) glycerol, 20 mg/mL DDM and 4 mg/mL CHS), supplemented with PIC and 1 mM PMSF. After 1 h at 20°C under gentle rotation, samples were ultracentrifuged at 125,000 *× g* during 1 h at 4°C, and the supernatant (detergent-solubilized proteins) were mixed with the home-made GFP-Trap^®^ beads previously equilibrated with 10 column-volumes of washing buffer (20 mM Tris-HCl pH 7.8, 150 mM NaCl, 100 mM KCl, 5 mM MgCl_2_, 20% (w/v) glycerol, 0.5 mg/mL DDM, 0.1 mg/mL CHS and 0.01 mg/mL POPS). After 2 h at 4°C of incubation with gentle rotation, the beads were washed with 20 column-volumes of washing buffer. GFP-Trap^®^ beads with bound PcATP2-GFP-BAD/Cdc50B-10xHis were diluted twice in washing buffer and flash-frozen in liquid-N_2_. The concentration of PcATP2-GFP-BAD in the beads was quantified by measuring the GFP fluorescence in a 96-well plate, and using purified GFP as reference. The protein content coupled to the GFP-Trap^®^ beads was eluted with 0.2 M glycine buffer pH 2.5 prior analysis by Coomassie-blue staining and western blot.

### ATPase assays

Between 5 and 10 µL of home-made GFP-Trap^®^ beads coupled with PcATP2-GFP-BAD/Cdc50B-10xHis (wild-type and mutant), and corresponding to 100 ng of PcATP2-GFP-BAD, were diluted into 50 µL of the reaction buffer (50 mM MOPS-Tris pH 7.0, 100 mM KCl, 5 mM MgCl_2_, 20% (w/v) glycerol, 1 mM NaN3, 0.5 mg/mL DDM, 0.1 mg/mL CHS), supplemented or not (“no lipid” experiments) with 0.45 mg/ml of the phospholipid substrate and, when indicated, with 0.025 mg/ml of PI4P. Samples were incubated at room temperature for 10 min and moved to ice for 15 min more. The reaction was initiated after the addition of 1 mM ATP-Mg^2+^ and incubated at 37°C for 25 min. The reaction was stopped by dropping the test tubes into liquid N_2_. To quantify the hydrolyzed ATP, we used the BIOMOL® Green solution (Enzo Biochem Inc), a proprietary mixing solution that uses a molybdate/malachite green-based assay to quantify the released Pi. Thus, 100 µL of BIOMOL® Green solution was added to each sample, vortexed and incubated at room temperature for 30 min. After incubation, samples were quickly spun down to pellet the beads, and 100 µL of clear supernatant were deposited in a 96-well plate to measure the absorbance at 620 nm. Standard calibration curves at each experimental buffer condition were made using a phosphate standard solution provided by the BIOMOL® Green supplier.

### PcATP2 3D model

To build the PcATP2 model, a divide and conquer strategy was taken. TOPCONS algorithm [34] was used to infer membrane protein topology, resulting in 10 TMs. The large intracellular domains (ICD) between TM2 and TM3 (ICD1) and TM4 and TM5 (ICD2) were aligned using MAFFT [35] and modeled independently in MODELLER [36]. ICD1 was modeled against templates 6KG7, 6ROH, and 1IWO. ICD2 was modeled against templates 6K7G, 6ROH, 1IWO, 4HQJ, and 5MPM. All TMs were modeled by carbon alpha molecular replacement in UCSF Chimera [37] using 6K7G and 6ROH as templates. The whole PcATP2 sequence was realigned using MAFFT and manually refined against the ICD1, ICD2, and the TMs. This alignment was used as template to build the overall PcATP2 model in MODELLER. PcATP2 model was embedded in an POPC bilayer, energy minimized, and equilibrated for 10 ns using the CHARMM36F [38] force field.

### Fluorescent confocal microscopy

1 mL of small-scale cultures was centrifuged for 5 min at 2000 *× g* followed by two washing steps with 1 mL of PBS pH 7.4. The cells were re-suspended in 1 mL of PBS pH 7.4, and 2 µL were deposited on an agarose pad (1% (w/v) agarose gel in milliQ water). The pictures were taken in the Light Microscopy Facility of ImaGif (I2BC, Gif-sur-Yvette, France) using a Leica confocal microscope SP8 controlled by the LAS-X software and using an oil immersion objective lens with 100 x magnification. Samples were excited with a 488 nm laser.

## Results

### ATP2 and Cdc50 proteins are highly conserved in *Plasmodium* species and present in other apicomplexan parasites

ATP2 is highly conserved in *Plasmodium* species. For instance, *P. falciparum* ATP2 (PfATP2, VEuPathDB ID: PF3D7_1219600), *P. vivax* ATP2 (VEuPathDB ID: PVX_123625), and the ATP2 orthologs from the malaria mouse models *P. berghei,* (VEuPathDB ID: PBANKA_1434800) and *P. chabaudi* (VEuPathDB ID: PCHAS_1436800, named in this work PcATP2) (Figure supplement 1) share ∼60% amino acid identity [8]. In addition, homologs to ATP2 are also present in the genome of other disease-causing intracellular parasites belonging, to the Apicomplexa phylum like *Plasmodium* (Figure supplement 1) [8], although it is still unknown if these transporters are also essential. These sequences contain the typical membrane topology of 10 predicted transmembrane segments (TMs), the conserved P-type ATPase motif, DKTG, (intracellular loop between TMs 4 and 5, where the aspartate is transiently autophosphorylated during the transport cycle), and the specific P4-ATPase motif DGET (intracellular loop between TMs 2 and 3), implicated in protein dephosphorylation [10]. Moreover, the well-conserved PICL (or PISL) motif present in TM4 of P4-ATPases is also conserved in these apicomplexan sequences (Figure supplement 1). Interestingly, the glutamine in TM1 and the two asparagine residues in TMs 4 and 6, that coordinate the substrate’s phosphatidylserine (PS) moiety in ATP8A1 [24] and in ATP11C [28], are also conserved in these apicomplexan sequences (Figure supplement 1). In ATP8A1, the isoleucine and leucine residues of the PICL motif in TM4 also stablish hydrophobic contacts with the PS moiety of the substrate [24].

The genome of *P. falciparum* encodes three Cdc50/LEM3 proteins: PfCdc50A (VEuPathDB ID: Pf3D7_0719500), PfCdc50B (VEuPathDB B ID: Pf3D7_1133300) and PfCdc50C (VEuPathDB ID: Pf3D7_1029400). Like ATP2, each Cdc50 protein has close homologs in other *Plasmodium* species (between 50–60% amino acid identity between *Plasmodium* species, depending on the Cdc50 protein, see as example the alignments of Cdc50B orthologs in Figure supplement 2), as well as in other apicomplexan parasites. All these sequences contain the two predicted TMs connected by a large extracellular (or luminal) domain, as well as the four well-conserved cysteines, known to participate in two disulphide bridges [23,24]. These disulphide bridges are important for the association of the Cdc50 protein with the P4-ATPase and for the activity of the complex [39].

### Screening *Plasmodium-encoded* ATP2 and Cdc50 proteins for *S. cerevisiae* heterologous production

To unravel the functional and structural features of PfATP2, we examined its heterologous expression together with its putative Cdc50 proteins in the yeast *Saccharomyces cerevisiae*. We screened for expression of the ATP2 and Cdc50 sequences encoded by three different *Plasmodium* species: *P. falciparum*, *P. berghei* and *P. chabaudi* aiming at identifying ATP2 and Cdc50 candidates with suitable expression yield for biochemical studies. For western blot detection, we fused a biotin acceptor domain (BAD) at either the N-terminal (BAD-ATP2) or the C- terminal (ATP2-BAD) ends of the ATP2 sequences, and a 10xHis-tag at the C-terminal end of each Cdc50 protein [31]. The *P. chabaudi* ortholog, PcATP2, was the only one that expressed in our system. The sequence similarity of ATP2 and Cdc50 ortologs in *Plasmodium* suggest similar functional and structural features, thus making PcATP2 a fair paradigm of PfATP2.

**Figure 1.**
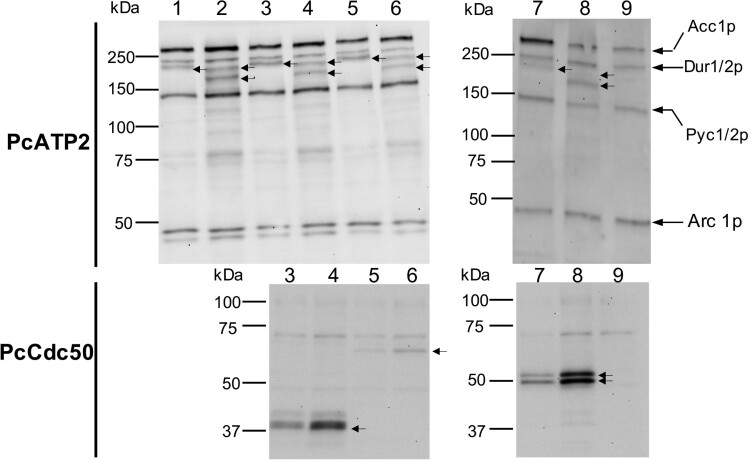
Analysis of PcATP2 expression in *S. cerevisiae* membranes, alone or co-expressed with each of the three putative PcCdc50 subunits. 5 µg of total protein were loaded on each lane. Top panels, western blots revealed with the probe against the BAD tag. Bottom panels, western blots revealed with the HisProbe^TM^ to detect the 10xHis tag. Gel bands corresponding to PcATP2 and the *P. chabaudi* Cdc50 subunits are indicated by arrows. *Lane 1*: single expression of PcATP2-BAD. *Lane 2*: single expression of BAD-PcATP2. *Lane 3*: co-expression of PcATP2-BAD and PcCdc50B-His. *Lane 4*: co-expression of BAD-PcATP2 and PcCdc50B-His. *Lane 5: c*o-expression of PcATP2-BAD and PcCdc50C-His. *Lane 6:* co-expression of BAD-PcATP2 and PcCdc50C-His. *Lane 7*: co-expression of PcATP2-BAD and PcCdc50A-His. *Lane 8*: co-expression of BAD-PcATP2 and PcCdc50A-His. *Lane* 9: empty Vector, membranes expressing no *Plasmodium* proteins (negative control). The theoretical molecular weight mass of PcATP2-BAD or BAD-PcATP2 is 180 kDa. The theoretical molecular weight masses of PcCdc50B-His, PcCdc50C-His and PcCdc50A-His are, respectively, 45, 61 and 51 kDa. Lanes 1–6 were run in a 8% acrylamide gel. Lanes 7–9 were run in a gel containing a gradient of acrylamide between 4–15%. Naturally occurring biotinylated *S. cerevisiae* proteins are indicated in lane 9: Acc1p, acetyl-CoA carboxylase; Dur 1/2p, urea carboxylase; Pyc 1/2p, pyruvate carboxylase isoforms 1 and 2, and Arc 1p, complex acyl-RNA[40].

Figure 1 shows the expression profiles of the two BAD-tagged versions of PcATP2, alone, or co-expressed with each of the three *P. chabaudi*-encoded Cdc50 proteins. *S. cerevisie* carries at least six different biotynilated proteins, and some of them are also detected in our western blots when using the Avidin HRP-conjugate probe (see control experiment in lane 9, and figure legend of Figure 1 for description)[40]. Nevertheless, the position of the bands of the different *S. cerevisiae* biotynitaled proteins did not overlay the ones of the BAD-fused PcATP2. PcATP2 displayed a slightly different electrophoretic mobility depending on the position of the BAD tag (Figure 1), previously observed in other P4-ATPases tagged as well with the BAD at either of the two extremities [31]. Also, BAD-PcATP2 displayed two electrophoretic bands, suggesting either a cleavage near the C-terminal end or two different electrophoretic mobilities of the same construct (Figure 1, lanes 2, 4, 6 and 8). Western blot analysis also revealed that the three putative PcCdc50 proteins were also co-expressed with both BAD-PcATP2 and PcATP2-BAD (Figure 1, bottom panels). We observed that the intensity of the electrophoretic mobilities of BAD-PcATP2 are influenced by the presence of the PcCdc50 proteins since the lower (or fastest) band became practically the only one when co-expressed with PcCdc50A (Figure 1, lane 8). PcCdc50A displayed two electrophoretic mobilities, suggesting protein glycosylation events typical of Cdc50 proteins (Figure 1, lanes 7 and 8). Overall, when either BAD-PcATP2 or PcATP2-BAD were co-expressed with each of the PcCdc50 proteins neither the electrophoretic pattern nor the intensity of the PcATP2 bands changed substantially. We discontinued working with PcCdc50C because of its low detection yield compared with the other two Cdc50 subunits.

**Figure 2.**
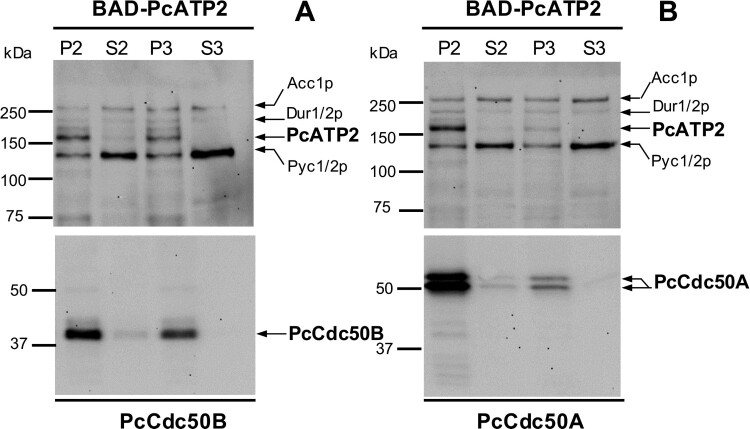
Analysis of membrane fractions of *S. cerevisiae* co-expressing PcATP2 with either PcCdc50B or PcCdc50A. Top panels, western blots revealed with the probe against the BAD. Bottom panels, western blots revealed with the HisProbe^TM^ to detect the 10xHis tag. (*A*) membrane fraction co-expressing BAD-PcATP2 and PcCdc50B-His. (*B*) membrane fraction co-expressing BAD-PcATP2 and PcCdc50A-His. The bands corresponding to BAD-PcATP2, PcCdc50B-His and PcCdc50A-His are indicated. P2 and S2 are, respectively, the membrane pellet and the supernatant obtained after centrifugation at 20,000 *× g*. P3 and S3 are, respectively, the membrane pellet and the supernatant obtained after centrifugation at 125,000 *× g*. Each lane was loaded with the corresponding membrane fraction or supernatant containing 1 µg of total protein. Biotinylated *S. cerevisiae* proteins are also indicated: Acc1p: acetyl-CoA carboxylase, Dur1/2p: urea carboxylase, and Pyc1/2p: pyruvate carboxylase isoforms 1 and 2[40].

We also examined the expression yield in two different membrane fractions obtained from differential centrifugation of BAD-PcATP2 co-expressed with either PcCdc50A-His or PcCdc50B-His. We detected the presence of PcATP2 and the two PcCdc50 subunits in both, the P2 and in the P3 membrane fraction (Figure 2A). Notably, PcATP2 changed its relative distribution between P2 and P3 depending on the co-expressed PcCdc50 subunit (Figure 2A and B). That is, while the relative amount of PcATP2 in P2 or P3 was similar when co-expressed with PcCdc50B (Figure 2A), PcATP2 seems to be mostly accumulated in P2 when co-expressed with PcCdc50A (Figure 2B). In addition, the relative distribution of PcATP2 between P2 and P3 followed the same relative distribution as its co-expressed PcCdc50 protein (Figure 2A and B, bottom panels).

**Figure 3.**
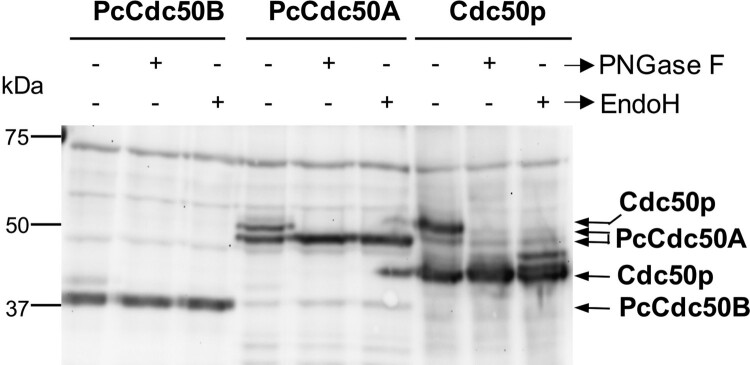
Deglycosylation assays of PcCdc50B and PcCdc50A co-expressed with PcATP2. Membranes co-expressing PcATP2/PcCdc50B, PcATP2/PcCdc50A or Drs2p/Cdc50p were enzymatically deglycosylated with PNGase F or EndoH, as indicated. 10 µg total protein were loaded in the experiments with PcATP2/PcCdc50B and PcATP2/PcCdc50A, whereas 5 µg of total protein were loaded in the control experiment with Drs2p/Cdc50p. The western blots were revealed with the HisProbe^TM^ to detect the 10xHis tag.

**Figure 4.**
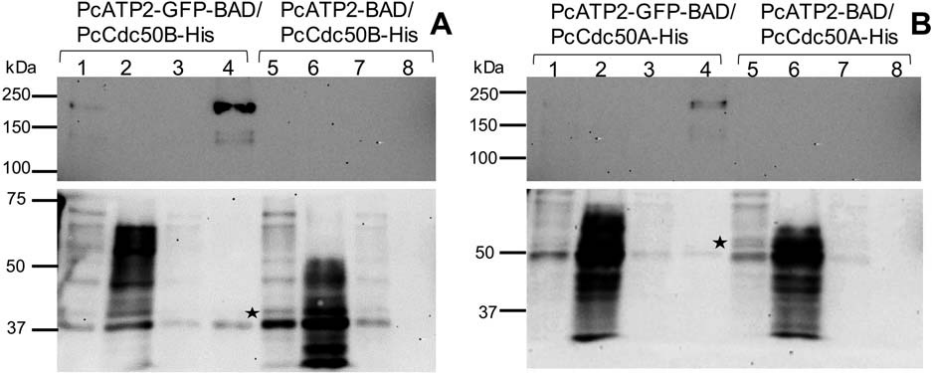
Co-immunoprecipitation of PcATP2-GFP-BAD with PcCdc50B-His or PcCdc50A-His. Lanes *1*–*4* correspond to the different steps of the assay with membranes co-expressing PcATP2-GFP-BAD and PcCdc50B-His (*A*) or PcCdc50A-His (*B*): (*1*) input, detergent-solubilized membranes, (*2*) flow-through or non-bound protein fraction, (*3*) washings, and (*4*) elution. Lanes *5*–*8* are equivalent to lanes *1*–*4* but with membranes co-expressing PcATP2-BAD and either, PcCdc50B-His (*A*) or PcCdc50A-His (*B*). The blots on the top panel were revealed with an antibody against the GFP to detect PcATP2-GFP-BAD, and the bottom one with the HisProbe^TM^ to detect PcCdc50B-His and PcCdc50A-His (labelled with an asterisk).

We screened a battery of eleven detergents to solubilize BAD-PcATP2 and the co-expressed Cdc50 proteins from both, P2 and P3 membranes. PcATP2 and Cdc50 proteins in P2 membranes could not be solubilized under any of the tested experimental conditions, with the exception of the rather denaturing n-dodecylphosphocholine (Fos-Choline-12). In contrast, a fraction of PcATP2 and PcCdc50B from P3 membranes was solubilized in N-dodecyl-β-D-maltopyranoside (DDM) and, with less efficiency, in Lauryl maltose neopentyl-glycol (LMNG) or Octaethylene glycol monodecyl ether (C12E8) (Figure supplement 3). The fact that only the PcATP2 and PcCdc50B fractions present in P3 could be solubilized in DDM strongly suggests a folding defect of the P2-containing proteins because DDM is know to be poorly efficient fo solubilize unfolded or partially folded integral membrane proteins[41]. Importantly, the solubilization efficiency of these detergents was greatly improved by the presence of the cholesterol derivative, cholesteryl hemisuccinate (CHS) (Figure supplement 3). Due to the low amount of BAD-PcATP2 co-expressed with PcCdc50A in P3 (Figure 2), the immunodetection of the DDM/CHS solubilized fraction of BAD-PcATP2 from P3 membranes was always challenging (Figure supplement 3, panel E). Interestingly, we found that the lower band of PcCdc50A (identified in the next section as the non-glycosylated form of PcCdc50A, Figure 3) was the only one co-solubilized with PcATP2 (Figure supplement 3, panel F or Figure 4, right panel, lanes 1, 4 and 5). This suggests that glycosylation of PcCdc50A (see next section) might affect the folding and/or membrane trafficking during biogenesis, explaining (at least, in part) the preferential localization of PcCdc50A together with its co-expressed PcATP2 partner in P2 membranes (Figure 2, right panels).

### N-Glycosylation of PcCdc50A and PcCdc50B expressed in *Sacharomyces cerevisiae*

As seen in Figures 1 and 2, PcCdc50A-His presented two electrophoretic bands, suggesting glycosylation events typical of Cdc50 proteins. Glycosylation of Cdc50 proteins plays often important roles in the stability and the activity of the P4-ATPase/Cdc50 complex [39,42]. Using the online server NetNGlyc (http://www.cbs.dtu.dk/services/NetNGlyc/), one potential glycosylation site was predicted at position N260 within the FNDT motif situated in the extracellular or luminal domain that connects TMs 1 and 2 of PcCdc50A (Figure supplement 4). This motif is conserved in Cdc50 proteins from others organisms, including the *S. cerevisiae* Cdc50 protein, Cdc50p, whose glycosylation state has already been observed when overexpressed in *S. cerevisiae* [23]. In contrast, no potential N-glycosylation sites were predicted in the extracellular or luminal sequence of PcCdc50B. To analyze the possible N-glycosylation of PcCdc50A and PcCdc50B when expressed in *S. cerevisiae*, we performed enzymatic deglycosylation assays using membranes co-expressing BAD-PcATP2/PcCdc50B-His or BAD-PcATP2/PcCdc50A-His, and using two deglycosydases: the amidase PNGase F and the N-glycosydase EndoH. As control, we used Cdc50p co-expressed with its *S. cerevisiae* P4-ATPase partner, Drs2p (BAD-Drs2p/Cdc50p-His). Consistent with previous studies [31], Cdc50p ran as two major bands in the western blot (Figure 3); the fully glycosylated fraction (∼50 kDa) and the non-glycosylated one (∼37 kDa). After incubation with either PNGase F or EndoH, the glycosylated band of Cdc50p disappeared while the intensity of the lowest non-glycosylated band increased. Similarly, the upper band of PcCdc50A disappeared after incubation with either PNGase or EndoH, while the lowest band became more intense with the same electrophoretic mobility (Figure 3), thus confirming the N-glysosylation of PcCdc50A. Conversely, the band corresponding to PcCdc50B remained unaltered after enzymatic treatment with regard both electrophoretic mobility and intensity (Figure 3). Of note, just above the band of PcCdc50B there is a faint band in the non-treated protein sample that disappeared after enzymatic digestion (Figure 3). This band could represent a non-specific protein since it is also present (although less intense) in the experiment with untreated PcCdc50A. Nevertheless, we can conclude from these experiments that most of PcCdc50B expressed in *S. cerevisiae* is not N-glycosylated.

### PcATP2 associates with PcCdc50B and PcCdc50A in detergent micelles

To assess the association of PcATP2 with PcCdc50B or PcCdc50A even after DDM/CHS solubilization, we performed immunoprecipitation studies. We fused the superfolder green fluorescent protein (GFP) at the C-terminal end of PcATP2 followed by the BAD (PcATP2-GFP-BAD). We chose to tag PcATP2 at the C-terminal end rather than the N-terminal like the previous experiments because the GFP is a more sensitive folding reporter when fused at the C-terminal end of the target protein [41]. The presence of both co-expressed proteins in P3 membranes was confirmed by western blot (Figure supplement 5, lanes 1 and 2). In addition, the relative distribution of C-terminally tagged PcATP2 between P2 and P3 membranes, and the solubilization efficiency of DDM/CHS of PcATP2-GFP-BAD/PcCdc50B-His or PcATP2-GFP-BAD/PcCdc50A-His from P3 membranes were very similar to the N-terminally tagged version of PcATP2 (not shown). Therefore, DDM/CHS-solubilized PcATP2-GFP-BAD/PcCdc50B-His or PcATP2-GFP-BAD/PcCdc50A-His were trapped in agarose beads coupled to an anti-GFP nanobody (GFP-Trap^®^), and bound proteins were eluted with a low-pH buffer and analyzed by western blot. As expected, PcATP2-GFP-BAD was detected in both elution fractions (Figure 4A and B, lanes 4 in top panels). Moreover, PcCdc50B-His and PcCdc50A-His also coeluted with PcATP2-GFP-BAD in their respective experiments (Figure 4A and B, lanes 4 in bottom panels), demonstrating that PcATP2 interacts with either PcCdc50B or PcCdc50A in the membrane and after detergent solubilization. The western blot signals of eluted PcATP2-GFP and PcCdc50A-His were much weaker than the ones of the PcATP2-GFP/PcCdc50B-His experiment, in concordance with their relative amounts in the P3 membrane fraction (Figure 2 and Figure supplement 5). As control, we performed the same experiment using P3 membranes co-expressing a non GFP-tagged PcATP2 with either PcCdc50B (PcATP2-BAD/PcCdc50B-His) or PcCdc50A (PcATP2-BAD/PcCdc50A-His). In this case, none of the PcCdc50 proteins were detected in the elution fraction (Figure 4, lane 8 bottom panels), thus excluding a false positive result due to a non-specific binding of PcCdc50B-His or PcCdc50A-His to the GFP-Trap^®^ beads.

**Figure 5.**
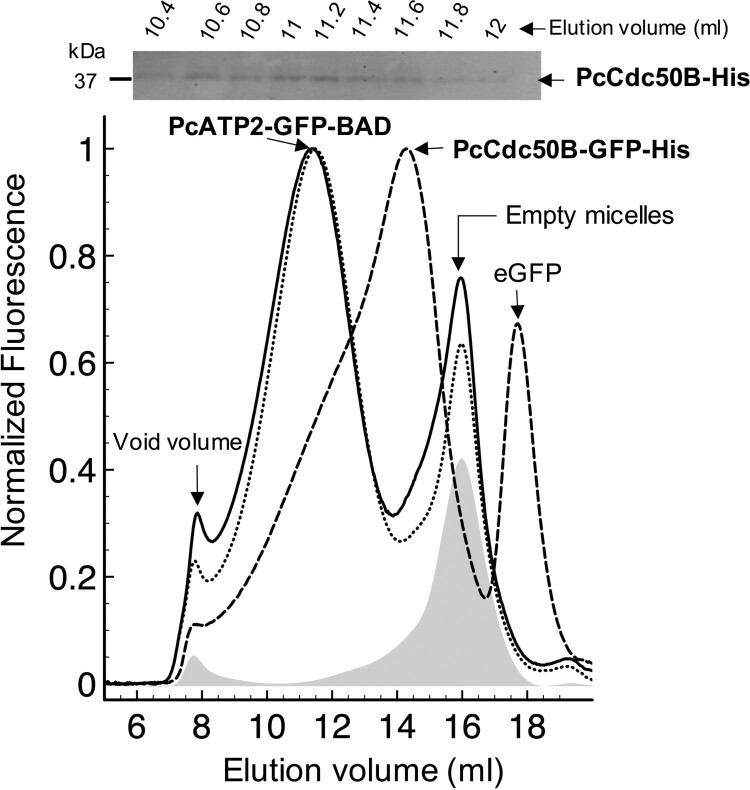
Fluorescence-detection Size Exclusion Chromatography of detergent solubilized PcATP2 and PcCdc50B. Membranes containing 5 mg/ml of total protein concentration were solubilized with 1% (w/v) DDM, 0.2% (w/v) CHS, and the supernatant after ultracentrifugation was loaded into a Superose 6 10/300 GL gel-filtration column equilibrated with 20 mM Tris-HCl pH 7.8, 150 mM NaCl, 10% (v/v) Glycerol, 0.1 mg/mL DDM, 0.02 mg/mL CHS, and connected to a fluorescence detector. Normalized FSEC profiles of single-expression of PcATP2-GFP-BAD (dotted lines) and co-expression of PcATP2-GFP-BAD with PcCdc50B-His (solid line). A profile of membranes expressing no protein is shown (in grey shadow). Normalized FSEC profile of single-expression PcCdc50B-GFP-His (dashed line). The presence of free eGFP is indicated. On top of the figure, analysis of PcCdc50B-His co-elution with PcATP2-GFP-BAD (solid line chromatogram). Collected fractions of 200 µl between 10.4–12 ml of the PcATP2-GFP-BAD elution were analyzed by western blot using the HisProbe^TM^ to detect the presence of PcCdc50B-His.

We also analyzed PcATP2 and PcCdc50B association by fluorescence-detection size-exclusion chromatography (FSEC). We used the GFP fluorescence to monitor the elution profile of DDM/CHS-solubilized PcATP2 in a gel-filtration column, and analyzed the degree of monodispersity (or stability) in detergent micelles of PcATP2 in the presence or in the absence of PcCdc50B. Detergent-solubilized PcATP2-GFP-BAD eluted as a nearly symmetric wide peak centred at ∼11.5 ml (Figure 5, dotted line). The second peak at ∼16 ml elution corresponded to the light-scattering of empty DDM/CHS micelles, visible in the chromatogram because the instrumental settings used to detect PcATP2-GFP-BAD. DDM/CHS-solubilized membranes expressing no GFP-tagged protein (shadow chromatogram in Figure 5) and the FSEC profile of 1% DDM 0.2% CHS confirmed this. PcATP2’s elution profile did not change significatively when PcCdc50B-His was co-expressed (Figure 5, solid line). However, the western blot analysis of the eluted fractions along the PcATP2-GFP-BAD elution peak confirmed that PcCdc50B-His co-eluted with PcATP2-GFP-BAD (Figure 5, upper panel) following a similar elution profile than PcATP2-GFP-BAD. Since the theoretical molecular mass of PcATP2-GFP-BAD and PcCdc50B-His are, respectively, 205 and 47 kDa, the co-elution of both proteins supports their association in detergent micelles. No significant shift in the retention time of PcATP2-GFP-BAD was observed when PcCdc50B-His was present (Figure 5, solid line), likely due to the low-resolution of the column at the range of molecular weights where PcATP2-GFP-BAD elutes. To discard the elution of free or non-associated PcCdc50B at the same retention time than PcATP2, we fused the GFP at the C-terminal end of PcCdc50B (PcCdc50B-GFP-His) and performed FSEC analysis of DDM/CHS-solubilized membranes expressing only PcCdc50B-GFP-His. The elution profile of PcCdc50B-GFP-His (Mw 76 kDa) in the absence of PcATP2 showed a main peak at ∼14.3 ml (dashed line in Figure 5), far from the PcATP2-GFP-BAD main elution (∼11.5 ml, dotted line Figure 5). Interestingly, the relatively broad and asymmetric shape of the chromatogram suggested a poor stability of PcCdc50B-GFP-His in DDM/CHS when PcATP2 is absent. Collectively, immunoprecipitation studies revealed the association of PcATP2 with either PcCdc50A or PcCdc50B. Moreover, FSEC experiments also confirmed the association of PcATP2 with PcCdc50B.

### Detergent-solubilized PcATP2/PcCdc50B hydrolizes ATP in the presence of POPS and POPE, and senses PI4P

**Figure 6.**
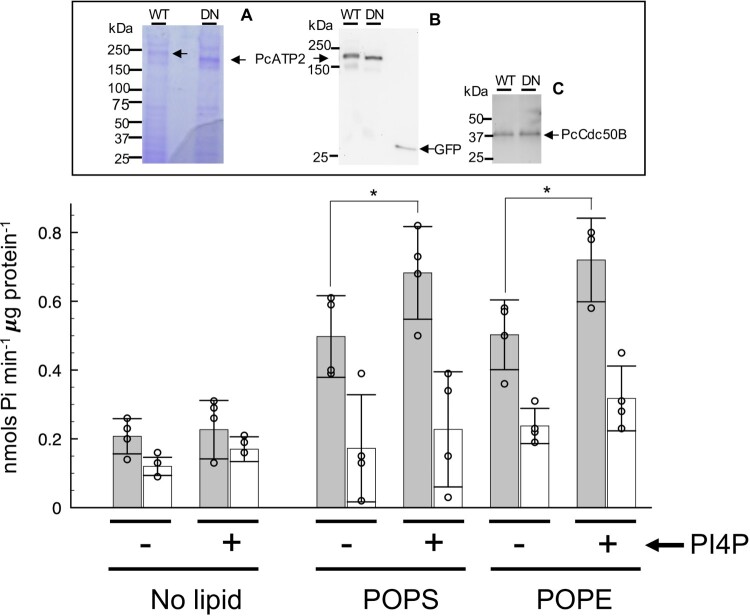
Lipid-stimulated ATPase activity of PcATP2/PcCdc50B complex. The apparent ATPase activity of the PcATP2/PcCdc50B complex was measured by spectrophotometric quantification of the released inorganic phosphate in the absence (no lipid) and in the presence of the indicated lipids. POPS, 1-palmitoyl-2-oleoyl-sn-glycero-3-phospho-L-serine, and POPE, 1-palmitoyl-2-oleoyl-sn-glycero-3-phosphoethanolamine. As indicated, ATPase assays were also performed in the presence (+) or in the absence (-) of phosphatidylinositol-4-phosphate (PI4P). Gray bars and white bars represent, respectively, the activity of the wild-type (WT) and the functionally-impaired D596N mutant (DN) at each experimental condition. The reaction was performed at 37°C during 25 min, and initiated by the addition of 1 mM ATP-Mg^2+^. The bars display the average value of *n *= 3 or *n *= 4 experimental datapoints, and the indicated error is the standard deviation. The upper panel displays the SDS-PAGE of the acid-eluted content of the immobilized wild-type (WT) or D596N mutant (DN) agarose beads used for this experiments. (*A*) Coomassie-stained, (B) western blot using the antibody against the GFP to detect PcATP2-GFP, and (*C*) western blot using the HisProbe^TM^ to detect the 10xHis tag fused to PcCdc50B .* *P *< 0.05 (paired t-test). Note that the DN mutant of PcATP2 runs slightly faster because it lacks the BAD domain.

In detergent micelles, P4-ATPases hydrolyze ATP only in the presence of their phospholipid substrates [43]. This property not only permits evaluating the activity of the transporter, but also it allows to assess substrate specificity. We assayed the functional properties of recombinant PcATP2-GFP-BAD/PcCdc50B-His complex by measuring the lipid-stimulated capacity of PcATP2 to hydrolize ATP. Detergent-solubilized PcATP2-GFP-BAD/PcCdc50B-His was immobilized in home-made GFP-Trap^®^ beads, and the liberated inorganic phosphate upon ATP hydrolysis was quantified spectrophotometrically after 25 min at 37°C in the presence of two putative phospholipid substrates. As negative control, we generated a predicted non-functional mutant of PcATP2 after replacing the aspartate residue at position 596 by an asparagine (Figure supplement 1). This aspartate is part of the conserved P-type ATPase motif DKTG, and undergoes phosphorylation and de-phosphorylation during each transport cycle [9]. Therefore, the resulting PcATP2-D596N-GFP mutant is expected to be fully deficient in ATP hydrolysis, as verified in all the tested P-type ATPases [9]. The presence of both PcATP2 variants and the co-expressed PcCdc50B in the GFP-Trap^®^ beads were confirmed by SDS-PAGE (Figure 6, upper panel). Although both PcATP2 versions were visible by Coomassie staining (Figure 6, upper panel, A), PcCdc50B was only visible after immunoblotting (Figure 6, upper panel, C). In addition, anti-GFP western blot analysis of the two PcATP2 variants eluted from the GFP-Trap^®^ beads confirmed the absence of protein degradation fragments containing the GFP (Figure 6, upper panel, B), thus validating the use of the GFP fluorescence to measure the concentration of PcATP2 in the beads. Immobilized PcATP2-GFP-BAD/PcCdc50B-His (called “WT” hereafter) in GFP-Trap^®^ beads displayed ATP hydrolysis in the presence of the phospholipids, POPS and POPE (grey bars in Figure 6), with an apparent ATPase activity between ∼0.5 and 0.7 nmols Pi min^−1^ μg protein^−1^. In the same conditions, the apparent activity obtained from the samples containing the functionally-impaired mutant PcATP2-D596N-GFP/PcCdc50B-His (called “DN” hereafter) ranged from ∼0.2 to 0.4 nmols Pi min^−1^ μg protein^−1^ (white bars in Figure 6). Moreover, in the absence of added lipids, the apparent activites of both WT and DN (“no lipid” in Figure 6,) were in the same range as the DN sample in the presence of POPS and POPE. The slightly higher apparent activity of WT with respect DN in the “no lipid” conditions (“no lipid” in Figure 6) is probably due to the presence of lipids carried by the protein during purification. Therefore, these results showed that the heterologously produced PcATP2 catalyzes ATP hydrolysis induced by the presence of the phospholipids POPS and POPE. The C-terminal end of PcATP2 and its *Plasmodium* orthologs contain the equivalent residues Tyr1235 and His1236 of Drs2p, recently identified as a phosphatidylinositol-4-phosphate PI4P recognition motif [23] (Figure supplement 6). In Drs2p, PI4P binding induces a strong increase of the substrate-stimulated ATPase activity [44,45]. In our experimental set up, the presence of PI4P slightly increased the signal background in all samples, since we observed a small increase in the average apparent activity in all control experiments (see the non-lipid assays and the DN assays in the presence of POPS and POPE), with increases of the apparent ATPase activity between 0.02 and 0.08 nmols Pi min^−1^ μg protein^−1^ (Figure 6). Nevertheless, we observed a larger increase of the POPS or POPE-stimulated ATPase activity of WT in the presence of PI4P (Figure 6). The net increase of WT ATPase activity, that is the difference of the apparent ATPase activities of WT in the presence and absence of PI4P after substracting the contribution of the DN sample at each condition were 0.13 and 0.14 nmols Pi min^−1^ μg protein^−1^ in the experiments with, respectively, POPS and POPE (Figure 6). In summary, the detergent-solubilized PcATP2/PcCdc50 complex produced in yeast is functionally competent as it catalyzes lipid-stimulated ATP hydrolysis in the presence of POPS and POPE, being also upregulated by PI4P.

## Discussion

In this paper we have demonstrated for the first time that purified PcATP2/PcCdc50B complex catalyses ATP hydrolysis in the presence of phospholipids. The essential and irreplaceable putative lipid flippase activity of ATP2 [4–6] confirms that lipid homeostasis is paramount for the parasite’s development, representing new opportunities for therapeutic intervention. There are obvious future possibilities since, for instance, PfATP2 was associated with drug-resistant phenotypes against two antimalarial drugs [20].

The challenge now is to understand why and when the transport activity of ATP2 and the other putative P4-ATPases are required within the complex life-cycle of the malaria parasite. The intracellular development inside the parasitized cell depends on an initial heavy supply of lipids to build the different membrane compartments, together with efficient mechanisms to ensure a suitable lipid composition on each membrane, as ∼75% of all lipids exhibit significant variations on its relative amount along parasite’s development, particularly during gametogenesis [46]. The *Plasmodium* parasite has a limited capacity of synthesizing fatty acids and phospholipids[46], and therefore, many lipids are scavenged from the host membrane or from the serum where P4-ATPases might have a relevant role.

**Figure 7.**
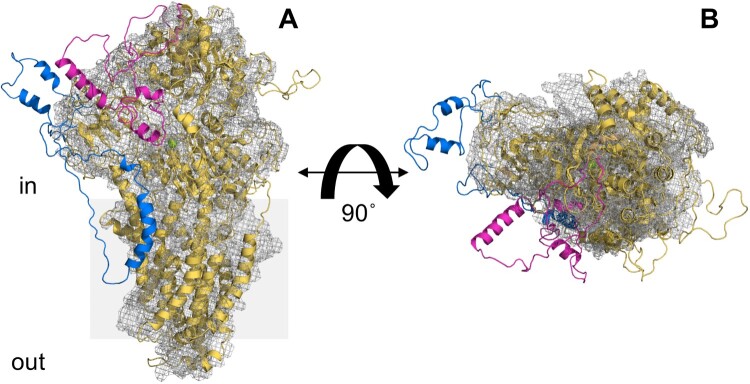
Alignment of the cryo-EM structure of Drs2p and a 3D structural model of PcATP2. The 3D structural model of PcATP2 (cartoon representation) was aligned with the cryo-EM structure of the autoinhibited conformation of Drs2p (PDB ID 6roh, mesh representation [23]). Protein domains of PcATP2 in the cytoplasmic region between TMs 2 and 3 that are absent in Drs2p are depicted in blue. Protein domains of PcATP2 in the cytoplasmic region between TMs 4 and 5 that are absent in Drs2p are depicted in magenta. (*A*) Lateral perspective of the molecule. (*B*) Cytoplasmic view after 90° rotation of *A* through the x axis. The grey shadow square in *A* represents the putative position of the surrounding membrane.

To unravel the structural and functional features of ATP2 we screened three ATP2 orthologs for heterologous expression in *S. cerevisiae*. In the present study we succeeded to express the *P. chabaudi* ATP2, PcATP2, a fair paradigm of *Plasmodium* ATP2 due to its high conservation in *Plasmodium* species (Figure supplement 1). This expression system was found to be successful in our group for PfATP6 [16], or for a yeast P4-ATPase [31]. The production of recombinant malaria MTPs is still very challenging, probably being one of the reasons for what 73% of *P. falciparum*-encoded MTPs are still considered as putative [7]. However, the use of recombinant proteins has been pivotal in malaria research, allowing the identification of substrates and inhibitors [47,48], providing protein sample for structural studies [49,50] or for confirming that PfATP6 is not an artemisinin drug-target [16]. One possible reason to explain the difficulty of expressing P-type ATPases encoded by apicomplexan parasites in heterologous hosts is that they contain non-conserved amino acid stretches within the two large cytoplasmic regions connecting TMs 2 and 3 and TMs 4 and 5 (Figure 7)[51], enclosing a high density of positively-charged and asparagine residues, a potential handicap for heterologous expression due to endoplasmic reticulum (ER) retention [52]. These extensions are absent in many other P-type ATPases, and as judged by a 3D model of PcATP2 based on the recent cryo-EM structure of Drs2p and ATP8A1 [23,24], they contain a large proportion of non-structured regions (Figure 7). Confocal microscopy images of *S. cerevisie* expressing PcATP2-GFP-BAD alone or co-expressed with PcCdc50B-His indicates that PcATP2-GFP-BAD is located in intracellular compartments (Figure supplement 7). This could indicate a possible retention of PcATP2-GFP-BAD in the ER as observed before in mammalian cell-lines for other P4-ATPase mutants presenting some folding defect [53]. Logically, in the absence of organellar markers we are unable to make further conclusions about the exact localization of PcATP2. Interestingly, a recent study showed that the *P. falciparum* K^+^ channel PfKch2 is also expressed in internal compartments of *S. cerevisiae* but it is active after detergent solubilization and purification[54].

Co-immunoprecipitation studies (Figure 4), together with the relative distribution of PcATP2 and PcCdc50A or PcCdc50B between P2 and P3 membrane fractions (Figure 2) and detergent-solubilization screenings (Figure supplement 3, panels *E* and *F*) demonstrated that the yeast-produced PcATP2 is able to associate with both PcCdc50B and PcCdc50A, two of the three *Plasmodium*-encoded Cdc50 isoforms. This promiscuous capacity to associate with more than one Cdc50 β-subunit is known in other P4-ATPases [10], although the physiological meaning of this dual interaction remains to be determined. We also found that *S. cerevisiae*-expressed PcCdc50A was N-glycosylated (Figure 3). In contrast, the majority of PcCdc50B expressed in *S. cerevisiae* seemed not to contain N-glycosylation (Figure 3). N-glycosylation is a typical signature of Cdc50 proteins [39], and its role seems to be organism-dependent. While in *Leishmainia infantum* Cdc50 glycosylation affects the catalytic activity of the P4-ATPase/Cdc50 complex [42], N-glycosylation of the Cdc50 β-subunit of the human ATP8A2 is required to form a stable complex with the P4-ATPases, with no apparent role on its catalytic activity [55]. Interestingly, the *P. yoeli* Cdc50A expressed in both gametocytes and ookinetes does not seem to be glycosylated as judged by the western blots bands ([29], and Jing Yuan, personal communication). Clearly, due to its potential functional and/or stabilizing role, further studies to unravel the glycosylation state of Cdc50A and Cdc50B in the parasite are necessary.

FSEC experiments confirmed the association of PcATP2 with PcCdc50B, as well as the stability of this complex in DDM/CHS detergent micelles (Figure 5). Our data also suggested that the cholesterol analog CHS is important to stabilize this complex since its presence highly improved the yield of co-solubilization of PcATP2 and PcCdc50B with DDM (Figure supplement 3). In the recent cryo-EM structure of the human ATP8A1 in complex with Cdc50A, a CHS molecule was found at the heterodimer interface between TMs 7 and 10 of ATP8A1 and TM2 of Cdc50A, suggesting a potential role of CHS on preserving the heterodimer [24]. Although *Plasmodium* parasites cannot synthesize *de novo* cholesterol, lipidomic studies have revealed the presence of this lipid in intracellular membrane compartments of infected erythrocytes [46], most likely obtained from the host cell-membrane.

The PcATP2/PcCdc50B complex produced in *S. cerevisiae* showed ATPase activity in detergent micelles, in the presence of the aminophospholipids POPS and POPE (Figure 6), thus demonstrating the capacity of PcATP2 to recognize these two phospholipids. Two amino acid clusters determine, at least in part, the lipid head-group selectivity of P4-ATPases [24]. In PcATP2, these clusters correspond to residues Gln65 and Leu66 in TM1, and Ala455 and Asn456 in TM4 (Figure supplement 1). PS-selective P4-ATPases normally contain polar residues at these two clusters (two Gln at the corresponding positions 65 and 66 of PcATP2, and two Asn at the equivalent positions 455 and 456 of PcATP2) that establish H-bonds with PS. In contrast, PC-selective P4-ATPases usually contain non-polar residues at the same positions [24]. PcATP2 has one polar residue at each cluster (Figure supplement 1) that, in principle, could be sufficient to coordinate PS, PE, and even PC. Although in the ATPase assays it is possible the presence of a non-functional fraction of PcATP2, it is also possible that PcATP2 is partially autoinhibited using a mechanism similar to its yeast homolog Drs2p [23,45]. In fact, the GFAFS motif at the C-terminus of Drs2p involved in enzyme’s autoinhibition by interacting with the nucleotide binding site [23], is also well conserved in PcATP2 (as well in the other *Plasmodium* ATP2 orthologs), although the length of the C-terminal of the *Plasmodium* sequences is ∼60 amino acid shorter (Supplementary Figure 5). In addition, PcATP2 shares with Drs2p a few residues in a ⁠short loop region of the nucleotide domain (residues 698–704 in Drs2p, see Supplementary Figure 5) that directly interact with the GFAFS motif to stabilize the autoinhibitory conformation [23]. Moreover, and as suggested by the presence of a PI4P binding motif on its amino acid sequence (Figure supplement 6)[23], PcATP2 is also upregulated by this lipid as we observed a statistically significant increase of POPS and POPE-stimulated ATPase activity in the presence of PI4P (Figure 6). PI4P is an essential lipid for the malaria parasite since the inhibition of PI4P synthesis in the Golgi apparatus completely blocks the late steps of the intraerythrocytic cycle of *P. falciparum* by interfering with membrane biogenesis around the developing merozoites [56]. PI4P is found at the plasma membrane and at the Golgi apparatus in all stages of the erythrocytic cycle [57]. Interestingly, the *P. berghei* ATP2 was also localized at the interface parasite/host [4]. It will be relevant to investigate if the lethal effects as a result of depleting PI4P or suppressing ATP2 are somehow connected and linked to the biogenesis of the parasite’s membranes.

In conclusion, our work provides the first insights into the function and regulation of a *Plasmodium* ATP2, as well as the identity of two Cdc50 β-subunits of this transporter. This information represents a first step towards the understanding of the essential role of this transporter during malaria infection, and towards its validation as antimalarial drug target.

## Supplementary Material

Supplemental Material
