# Supplementary material for: ATP2, The essential P4-ATPase of malaria parasites, catalyzes lipid-stimulated ATP hydrolysis in complex with a Cdc50 β-subunit
Source: Emerg Microbes Infect. 2021 Jan 17;10(1):132–47. doi: 10.1080/22221751.2020.1870413 (PMC7832587; doi:10.1080/22221751.2020.1870413)
Supplement: Supplemental Material [file TEMI_A_1870413_SM4042.pdf]

ATP2, the essential P4-ATPase of malaria parasites, catalyzes lipid-stimulated ATP hydrolysis in complex with a Cdc50 β-subunit

Anaïs Lamy[1,2], Ewerton Macarini-Bruzaferro[1,3], Thibaud Dieudonné[1,4], Alex Perálvarez-Marín[5], Guillaume Lenoir[1], Cédric Montigny[1], Marc le Maire[1] and José Luis Vázquez-Ibar[1,*]

[1]Université Paris-Saclay, CEA, CNRS, Institute for Integrative Biology of the Cell (I2BC), 91198, Gif-sur-Yvette, France. [2]Department of Medical Biochemistry and Biophysics, Umeå University, Umeå, Sweden. [3] Department of Clinical Medicine (FMUSP), University of São Paulo São Paulo, Brazil.[4] DANDRITE, Nordic EMBL Partnership for Molecular Medicine, Department of Molecular Biology and Genetics, Aarhus University, Aarhus, Denmark. [5]Biophysics Unit, Department of Biochemistry and Molecular Biology, School of Medicine, Universitat Autònoma de Barcelona, 08193 Cerdanyola del Vallés, Catalonia, Spain.

[*]Correspondance to : José Luis Vázquez-Ibar: jose-luis.vazquez-ibar@i2bc.paris-saclay.fr.

SUPPLEMENTARY FIGURES

Figure supplement 1

```
                                         TM1                TM2
P. falciparum       FHKISNVYFFFIGILQVIPQFTATNGIPTVFFPLLIVLTANAIKDAFEDWNRHKTDKIEN 116
P. vivax            FHKISNVYFLIIGILQLVPEFTATNRLPTILFPLTIVLVANAIKDAYEDWNRHKTDKIEN 116
P. berghei          FHKISNIYFFIIGVLQLVPELTATNRIPTILFPLSIVLIANAINDAYEDWNRHRTDKIEN 109
P. chabaudi         FHKISNVYFFIIGVLQLVPELTATNRIPTILFPLSIVLIANAINDAYEDWNRHKTDKIEN 109
T. gondii           FHKVSNVYFVVICCLQMIPQISTTNGVPTLALPLSIVLVVNAAKDAFEDWQRHRSDRIEN 109
C. parvum           FCRPVNFYFLVISLLQIFPSISSTNGIPTLALPLVFVLFVGAVKDGWEDLNRHQNDRIEN 115
B. microti          LKQPLSLYFLAIAILQITPSISATRGIPTVMLPLFIVIAIDSIKDAYEDWQRHTSDRAEN 112
                    : :  ..**. *  **: *.:::*. :**: :** :*:  .: :*.:** :** .*: **
-----------------------------------------------------------------------------------
P. falciparum       IAFVETSSLDGETNLKVKEANTFLFNILGNDRNSAIDNVKNLKGFILSDKPNKDLSTMYG 288
P. vivax            IAFVETSSLDGETNLKVKEANGFVFNILTSDRGEAIEKVKNLKGFIISEKPNKDLTTMYG 287
P. berghei          ICFAETSSLDGETNLKVKEVNKYIFNNLTYNMDEAIEKAKKLRGYILSEKPNKNLSTMNG 288
P. chabaudi         ICFAETSSLDGETNLKVKEVNKYIFNNLTYNIDEAIEKVKKLRGYILSEKPNKNLSTMNG 283
T. gondii           GAFVETASLDGETNLKLKQTHRVTFEWLGSFLPLAVCYLLTRAGRIRCQVPNRDLNTYEG 273
C. parvum           DVFIDTSSLDGESNLKRRFSHKESTKMLGNNIHDVIKRARYLEGLIECSPPGKDLHNFDG 276
B. microti          ISYVETLCLDGETNLKRKEAVQITQNYLKNDLINVLERIKNCEASILCNVPDTDLDKFKG 251
                    : :* .****:*** :        : *      .:        . * .. *. :* .  *
-----------------------------------------------------------------------------------
                                                   TM4
P. falciparum       PFNLE---------KAKKPYIVGIISFFSWVVITGNFVPISLIVTMSFVKVVQAYFISCD 518
P. vivax            PFNLE---------ESKKPFIVGVISFFSWVVITANFIPISLIVTMSFVKVVQAYFISCD 496
P. berghei          PFNLV---------EPKAPIISGIVSFFSWIVITANFIPICLIVTMSFVKVVQAYFISCD 490
P. chabaudi         PFNLV---------EPKAPIVSGIISFFSWIVITANFIPICLIVTMSCVKVIQAYFISCD 480
T. gondii           -TAAG---------NSEGPVVFCILNFFTWMVLTCNLVPISLVLQMGMVKALQSLFIAQD 453
C. parvum           GVSDVNEISYRATGQAIPISFVPVVRFCTWIVLLANIIPIALVVSMKIVKAIQGQFISRD 447
B. microti          -SPFY---------KDVTEVRVVCTSFFTWISITCNVIPISAVVTMNLVRFIQGYFISVD 409
                                  :           * :*: :   *.:**. :: *   *: :*. **: *
-----------------------------------------------------------------------------------
P. falciparum       ELGQIEYIFSDKTGTLTCNIMEFRKCAINGISYGKGLTEIKRNILKKKNLEIPVEPTM-K 708
P. vivax            ELGQIEYIFSDKTGTLTCNVMEFRKCAINGISYGNGLTEIKKHILKKKNMAIPEEPVL-K 684
P. berghei          ELGQIEYIFSDKTGTLTCNVMEFRKCAINGISYGTGLTEIKRKILKKNNIPIPQEPVDFD 656
P. chabaudi         ELGQIEYIFSDKTGTLTCNVMEFRKCAINGISYGTGLTEIKRKILIKNNIPIPPEPVDLD 645
T. gondii           ELGQVSYIFSDKTGTMTSNVMEFRKCCVRGLSYGQGLTEVRRQALRRLGLPVPADPLPPP 933
C. parvum           DLGQVRYIFSDKTGTLTRNIMEFKSLSVGGVHYGSTETSSSKEDNLIREIEIPQ------ 522
B. microti          LLGQVQICFSDKTGTLTCNKMNFRKFSIEGVSYGKGLTDIKRSYLIKNGIPVPGAISG-K 489
                    ***:    *******:* * *:*:. .: *: **    *.  :        : :*
-----------------------------------------------------------------------------------
                                  TM6
P. falciparum       QKIYFEFLLHLFNVLFTAIPVVIHAVLDQDISLNTAMEKPNLYKLGIHHYYFNIRTFISW 1359
P. vivax            QKIYYEFLLHLHNILFTAVPVVAHAILDKDVSLNTALVTPSLYKLGIYHYYFNISTFVSW 1318
P. berghei          QKIYYEFLLHLYNVMFTSLPIVILAILDKDVSLNTALKNPCLYKLGIHNFYFNINKFISW 1321
P. chabaudi         QKIYFEILLHSYNVLFTSLPIIILAILDKDVSINTALKNPCLYKLGIHNFYFNINKFISW 1288
T. gondii           QKFYFEFLYQMYNVVFTAIPITLYGVFDQDVDKKLALKYPQLYRCGQIDLYLNLRVFLKW 1463
C. parvum           TRLYFDYWYQVYNVILSSVPIVVVSVFDFDVTKSESLSKPHLYSFGPENKFLNTKICLIY 1155
B. microti          QLLYNDMLQQLFNIFFTAIPSIIFGSIEQDVDRNVTFKYPQLYKLGHINFYMNMRAFLTW 1017
                    :* :    : .*:.::::*     . :: *:  . ::  * **  *  . ::*     : :
```

Multiple sequence alignments of ATP2 homologs encoded by Apicomplexan parasites. The figure only shows the regions where conserved residues or motifs mentioned in the main text are located. Lines above define the predicted transmembrane segments (TM) of the *P. falciparum* ATP2 (PfATP2) calculated using TOPCONS [1]. Conserved regions of these sequences specific to P-type ATPases or P4-ATPases are framed. Residues involved in the coordination of the PS head group in the human ATP8A1 [2] are highlighted with a grey background. Eukaryotic Pathogen, Vector & Host Database Resources (VEuPathDB) ID codes: **PF3D7_1219600**: *P. falciparum* ATP2 (PfATP2); **PVX_123625**: *P. vivax* ATP2; **FBANKA_1434800**: *P. berghei* ATP2; **PCHAS_1436800**: *P. chabaudi* ATP2 (named PcATP2 in this work); **TGME49_247690**: *Toxoplasma gondii* ATP2 homolog; **cgd7_1760**: *Cryptosporidium parvum* ATP2 homolog, and **BMR1_01G01915:** *Babesia microti* ATP2 homolog. (*) indicates positions which have a single, fully conserved residue, (:) indicates conserved residues of strongly similar properties, and (.) indicates conserved residues of weakly similar properties.

Figure supplement 2

```
                                            TM1

P. falciparum   ------ERVVGPVWINKYSSMIYFLMFLFILNLSVGILILILSSKYIECRIPYEYKG--E 90
P. vivax        ------EKVIGPIWVPTYCSIIVFLLFLFFFNLLVGVAILIISSNYIECRVPYEYKS--Q 90
P. berghei      ------EKIFGPVFVYKYSTLIAFFIFLFILNLSIGIAILYLSSQYIECKIPYEYKS--Q 93
P. chabaudi     ------EKVFGPVFVYKYSTSIVFFIFLFILNLSVGIAILYLSSQYIECKIPYEYKT--Q 93
T. gondii       --QVHQEAGNGMYPLWSAGVVLRLCLLGALFFVSVGAWLIFEDEQHVECKLNYAEKTLQE 221
C. parvum       NKVI--NNIERWIPFYTPHYLILIYIFVGITFITVGIFLQIFSNNTIECIINYEDSPG-N 137
B. microti      VKILEWDIRDGVYMQRRSAPILILFIFILAINICISSLLWTRKVNFVECEIPYHQQP--V 145
                      :              : : ::     : :.  :   . : :** : *  .

P. falciparum   TFTKYSIVKVTPEQCKGQ----KNLKELNG-NINVHYEILGMQQNHYKFVSGMKKEQLNG 145
P. vivax        AYTKYSIVKVTPEHCKGN----ENLKQLKG-PINIHYEIYGVQQNHYRFLTSFKKEQLRG 145
P. berghei      PYTKYSIIKVTPEHCKGR----ENLKELKG-KINVHYEIYGVQQNHYSFMKSFNAEQIGG 148
P. chabaudi     PYTKYSIIKVTPEHCKGH----ENLKELKG-EINVHYEIYGVQQNHYSFMKSFNTKQLGG 148
T. gondii       GSSRYLLKGISSAHCTRE------VNELKGEEISVYAEMGHFFQNDAQVLWSRNDRQLAG 275
C. parvum       GKVIDTIVEIKSEHCNPSMINGNELKYLKG-DFFIYYQLRNFYQNNKSFIFSRSDRQLSG 196
B. microti      GNPTFVTIKVTHKECNKD----DKFALLEADDIFVYYKITNYPHLESSLSNGIVQEQLAG 201
                         :.  .*.        .  *:.  : :: ::    : .  .  .   .*: *

P. falciparum   NIFLKKEELEECYPLITFSEGKKKKKLLHPCGIFPWNVFTDSYIFYDKEPDEVPFP---T 202
P. vivax        DLFLQEKELSECFPLITYEQSG-TRKILHPCGILQWNVFTDSYIFYDKEPDESPFP---T 201
P. berghei      GIDVYKHDLNQCYPLITYFKDR-INKILHPCGILPWSVFTDNYIFYDKEPDDAPFP---D 204
P. chabaudi     KIFVSKDYLNHCYPLITYFKDR-INKILHPCGVLPWHVFTDNYIFYDKEPEDAPFP---D 204
T. gondii       KIFTDPKDVRECEPLATAVVGN-VTKVLHPCGALAWAVFTDKYQFLEGTPEGDNDQVPMK 334
C. parvum       ELIYNEETLSDCYPVIKDKQ----GKIFYPCGVATLTIFNDTFTILDGQN---------D 243
B. microti      NVISDSKQLHNCAPLDSIEHKG-VKKILHPCGIHAWNVFNDKIRFYRSSPTGS-LA---A 256
                 :    . : .* *: .        *:::***     :*.*.  :

P. falciparum   PLPLKQNVEEITI-KYYRQFYKNPSPQNVQLYKDHIYFWMEPDIQYERLQ-ENKETNEKL 260
P. vivax        PLPLKQRAEDITI-KYYRKFFKNPTRDIINLHKKRIYFWMDEEVQLKILQ-EHAETNDKL 259
P. berghei      PLPLNERVEDITI-KYFRKFFKNPHPENIKLYKDKVYFWMDAKTQSEALH-ENIVANEKL 262
P. chabaudi     PLPLKERVEDITI-KYFRKFFKNPHPETIDLYKDKVYFWMDRTTQSEALH-ENIVANEKL 262
T. gondii       PIPLNQTQAVLLHSWPWQDMYKNPPAEDRAAVLDKVYFWMSPVDNDDGEDMYKTREEARA 394
C. parvum       PIEIDDSIDTITF-KSDQINYKNIPEHELLNH---------------------------- 274
B. microti      SIEIDESVPTSAM-PLEIQHFKNPTQDIVDKHKQHTYFWMLPENEDSKEM-DDDEC--LA 312
                 : :.:              :**   .

                                                 TM2

P. falciparum   IND---TKIIVISTSQYYMRTF-LIGFIFIIISIIALILCIFYLIRMNKYENK------- 366
P. vivax        ISD---TKIIVISNADFYFNTT-LIGIFFIITAVFALLLSLLYFIRMKKHQFK------- 365
P. berghei      TAE---AKAIIITEANFYINNN-LIGIIFTIISIFSLILSILYYMRMKKHKFMRQI-DE- 373
P. chabaudi     TAE---AKAIIITEANFYINNN-LIGIIFIIISIFSLILSILYYMRMKKHKFMRRI-ED- 373
T. gondii       VSSWKGKKAIVLVQKSRFGGRSLFIGIAYLSFGCLLTM--LVFYMLWKKWQYRREGEEIR 509
C. parvum       VHFFNGSKHIVISQSTIFGGKNPYFGILYIISGILFILLSIYYIIRNKFNTNILG--DFR 391
B. microti      VAKFNGTKSIILSIPRWPYGSSLSLEILHLVFTILTLLFTVIYATRNTNSSTFLQMYHES 429
                       * *::            : : .     :  :  : :    .
```

Multiple sequence alignments of *Plasmodium* and Apicomplexan Cdc50B homologs. The figure only shows the regions where conserved residues or motifs mentioned in the main text are located. Lines above define the predicted transmembrane segments (TM) of the *P. falciparum* Cdc50B (PfCdc50B) calculated using TOPCONS [1]. The residues highlighted with a grey background correspond to the four conserved cysteines involved in the formation of the two disulfide bridges of the Cdc50 ectodomain. Eukaryotic Pathogen, Vector & Host Database Resources (VEuPathDB) ID codes: **PF3D7_1133300**: *P. falciparum* Cdc50B (PfCdc50B); **PVX_092270**: *P. vivax* Cdc50B; **PBANKA_091510:** *P. berghei* Cdc50B; **PCHAS_093090**: *P. chabaudi* Cdc50B (named PcCdc50B in this work); **TGME49_230820:** *Toxoplasma gondii* Cdc50B homolog; **cgd5_360**: *Cryptosporidium parvum* Cdc50B homolog, and **BMR1_03g01157**: *Babesia microti* Cdc50B homolog. (*) indicates positions which have a single, fully conserved residue, (:) indicates conserved residues of strongly similar properties, and (.) indicates conserved residues of weakly similar properties.

Figure supplement 3

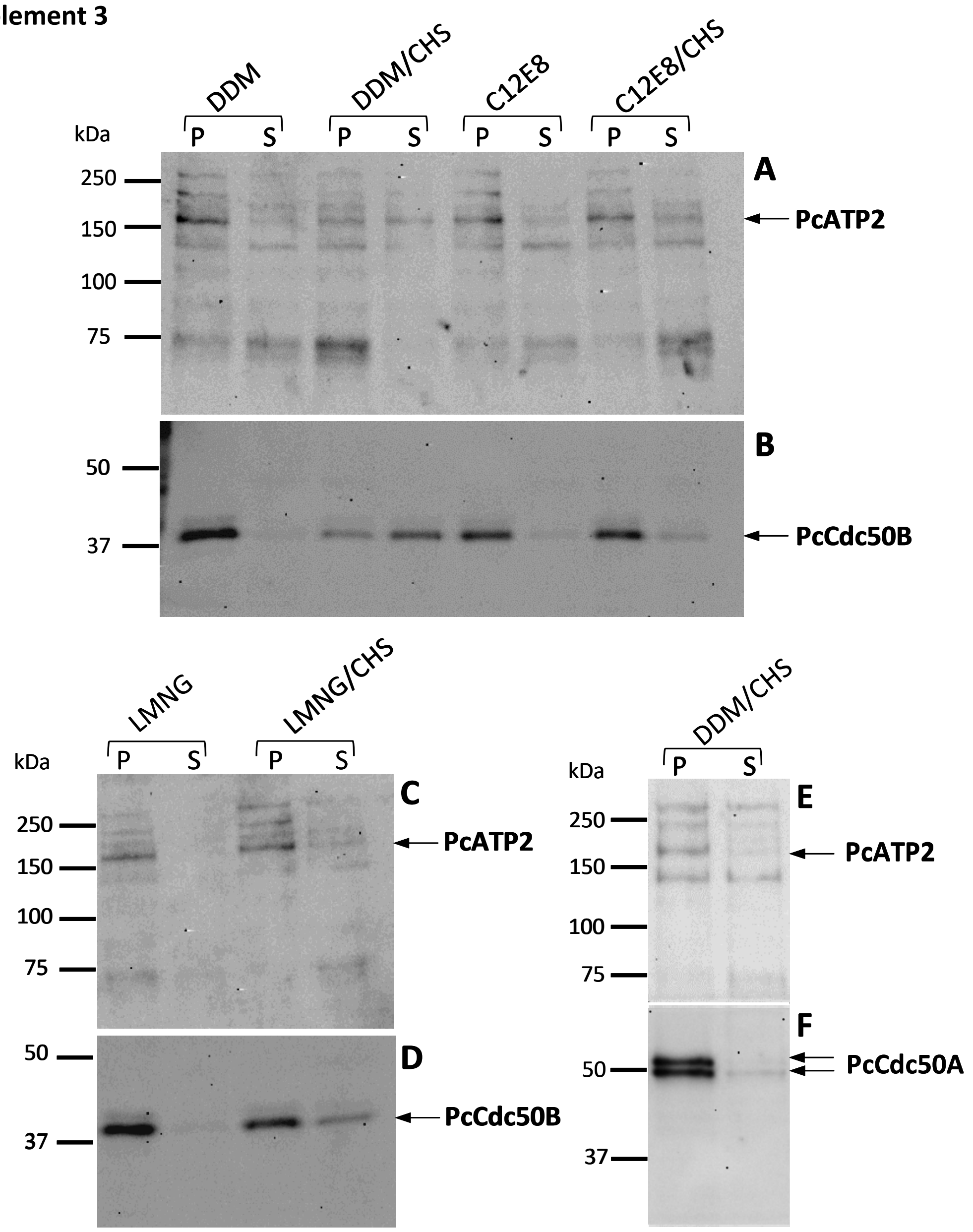

Detergent solubilization of P3 membranes co-expressing PcATP2 with either PcCdc50A or PcCdc50B. Membranes co-expressing BAD-PcATP2/PcCdc50B-His (panels *A* to *D*) or BAD-PcATP2/PcCdc50A-His (panels *E* and *F*) at 2 mg/ml of total protein concentration were solubilized in 1 % (w/v) of the indicated detergent for 1 h at 20°C, in the presence or absence of 0.2 % (w/v) of cholesteryl hemisuccinate (CHS). After ultracentrifugation, 1 µg of total protein of the pellet (*P*, non-soluble material) and the supernatant (*S*, soluble material) were loaded on each lane. Panels *A*, *C* and *E*, western blots revealed with the probe against the BAD. Panels *B*, *D* and *F*, western blots revealed with the HisProbe™ to detect the 10xHis tag. *DDM*, N-dodecyl-β-D-maltopyranoside, *LMNG*: Lauryl maltose neopentyl-glycol, C12E8, Octaethylene glycol monodecyl ether.

Figure supplement 4

```
P. falciparum   FYELHNFYQNHKKYLVSKSHNQLMGTVYTKDNEVSQCGPITKNHEGKILHPCGLIARSIF 291
P. vivax.       YYELHNFYQNHKKYLISKSHSQLMGTVYTRPDDLAQCFPITQNKEGKVLHPCGLVARSVF 262
P. berghei.     YYELHNFYQNHKKYLISKSQNQLMGVVYTNPSDISQCFPIITNKEGKILHPCGLVARSVF 253
P. chabaudi     YYELHNFYQNHKKYLISKSHNQLMGVVYTKASDVSQCFPIVTNKEGKVLHPCGLVARSIF 259
                :**************:***:.****.***. .:::** **  *:***:******:***:*

P. falciparum   NDTFSVYMDRELHNMIKLDESKEGITWYSDYNKFKNPSDSEMELHKSHVDFWLMNEKYKN 351
P. vivax.       NDTFTLYKHKTHSDRIEIDESKEAITWHSDLNKFKNPSEQQMKDHKEDVDFWLMNQNYVS 322
P. berghei      NDTFTLYKDVNLKEKIKIDESKEAIIWNSDYNKFKNPSEKEMNMYKESVYFWLTDKQYVD 313
P. chabaudi     NDTFTLYKDINLREKIKIDESKESIIWNSDYNKFKNPSKEEMDMYKESVYFWLNDKRYVD 319
                ****::* .    : *::*****.* * ** *******..:*. :*. * *** ::.* .
```

Conservation of a predicted N-glycosylation site in *Plasmodium* Cdc50A homologs. The figure only shows the aligned regions where conserved residues or motifs mentioned in the main text are located. The residues highlighted with a grey background correspond to a conserved N-glycosylated sequence [3,4], situated in the extracellular or luminal domain of the protein between TMs 1 and 2. Eukaryotic Pathogen, Vector & Host Database Resources (VEuPathDB) ID codes: **PF3D7_0719500**: *P. falciparum* Cdc50A (PfCdc50A); **PVP01_0315200**: *P. vivax* Cdc50A; **PBANKA_061700:** *P. berghei* Cdc50A; **PCHAS_061870**: *P. chabaudi* Cdc50A (named PcCdc50A in this work). (*) indicates positions which have a single, fully conserved residue, (:) indicates conserved residues of strongly similar properties, and (.) indicates conserved residues of weakly similar properties.

Figure supplement 5

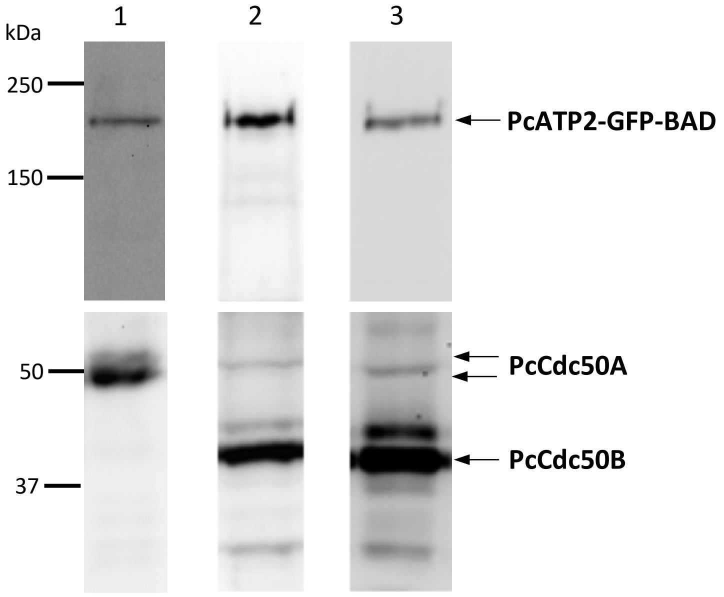

Western blot analysis of P3 membranes co-expressing PcATP2-GFP-BAD (wild-type and D596N mutant) with PcCdc50B-His, and PcATP2-GFP-BAD with PcCdc50A-His. Lane 1, co-expression of PcATP2-GFP-BAD with PcCdc50A-His; Lane 2, co-expression of PcATP2-GFP-BAD with PcCdc50B-His; Lane 3, co-expression of D596N-PcATP2-GFP-BAD with PcCdc50B-His. Top panels, western blots revealed with an antibody against the GFP to detect PcATP2-GFP-BAD. Bottom panels, western blots revealed with the HisProbe™ to detect the 10xHis tag of PcCdc50 proteins.

Figure supplement 6

```
P. falciparum ATP2 KHFDITFLYRREGKYGISIFG--KIYEIDTLATIEFTSKRKMSSVICRIPVINPDYNHPT 835
P. vivax ATP2      KHFGITFLYRRDGKYGISIFG--TVYEIETLAIVEFTSKRKMSSVICRIPVRATHTGGAG 811
P. berghei ATP2    KHFGITFLYRKDGKCGIKIFD--KVYEIDILATVEFTSKRKMSTVVCRIPIISNESTEPS 782
P. chabaudi ATP2   KHFGITFLYRKDGKCGVKIFD--KVYEIDILATIEFTSKRKMSTIVCRIPVMSNEDTKTS 771
Drs2p              ADLGYKFIIRKPNSVTVLLEETGEEKEYQLLNICEFNSTRKRMSA--------------- 707
                    .:. .*: *: ..  : :       * : *   **.*.**  :
--------------------------------------------------------------------------------
                              TM10
                    _____________________
P. falciparum ATP2 RFWLVVILGLFTALLRDYVFKVYKRNFNPEIYHLLLDQENAKIGMNDVIDQLKLNEFDKD 1522
P. vivax ATP2      RFWLVVLLGLFTALSRDFIFKVFKRNFNPEVYHFLLDQEDKPKGENNVINPLSSDPCQKE 1481
P. berghei ATP2    RFWLVLLLVLFTALTRDYVYKVYKKNFYPEAYHLLQDEEENISNTNHIQHS--SKCSNNM 1482
P. chabaudi ATP2   RFWLVFVLGLFAALTRDYVYKVYKKNFCPEAYHLLQDEEDKIENPKNIQHN--SRSSNNI 1449
Drs2p              VFWLTLIVLPIFALVRDFLWKYYKRMYEPETYHVIQEMQKYNISDSRPHVQQFQNAIRKV 1263
                    ***..::  : ** **:::* :*: : ** **.: : :.   . .            :

P. falciparum ATP2 DDIRIEKSKSLGYAFSEADPACIQLIRK-QDNMI-------------------------- 1555
P. vivax ATP2      EEIKIEKCKSLGYAFSEVDPACVKLIRK-QDKLI-------------------------- 1514
P. berghei ATP2    DETKSSKSEFMGYAFSEADPACVHFIRK-QDKLI-------------------------- 1515
P. chabaudi ATP2   EEMKPSKSELMGYAFSEADPVCVNFIRK-QDKLI-------------------------- 1482
Drs2p              RQ-VQRMKKQRGFAFSQAEEGGQEKIVRMYDTTQKRGKYGELQDASANPFNDNNGLGSND 1322
                    :      :  *:***:.:    . * :  *.
```

Conservation in *Plasmodium* ATP2 orthologs of the PI4P binding-site and the autoinhibitory domains of Drs2p. The figure shows the partial sequences alignments of *Plasmodium* ATP2 sequences and the *S. cerevisiae* P4-ATPase, Drs2p. Lines above define the predicted transmembrane segments (TM) of the *P. falciparum* ATP2 (PfATP2, PF3D7_1219600) calculated using TOPCONS [1]. Residues at the C-terminal end involved in the binding of PI4P in Drs2p [5], and also conserved in the *Plasmodium* sequences are highlighted with a grey background. Protein motifs in Drs2p involved in protein autoinhibition, EFNSTRK at the nucleotide domain and GFAFS at the C-terminal, are framed [5]. Eukaryotic Pathogen, Vector & Host Database Resources (VEuPathDB) ID codes: **PF3D7_1219600**, *P. falciparum* ATP2 (PfATP2); **PVX_123625**, *P. vivax* ATP2; **PBANKA_1434800**, *P. berghei* ATP2; **PCHAS_1436800**, *P. chabaudi* ATP2 (named PcATP2 in this work). Drs2p is a P4-ATPase of *S. cerevisiae*. (*) indicates positions which have a single, fully conserved residue, (:) indicates conserved residues of strongly similar properties, and (.) indicates conserved residues of weakly similar properties.

Figure supplement 7

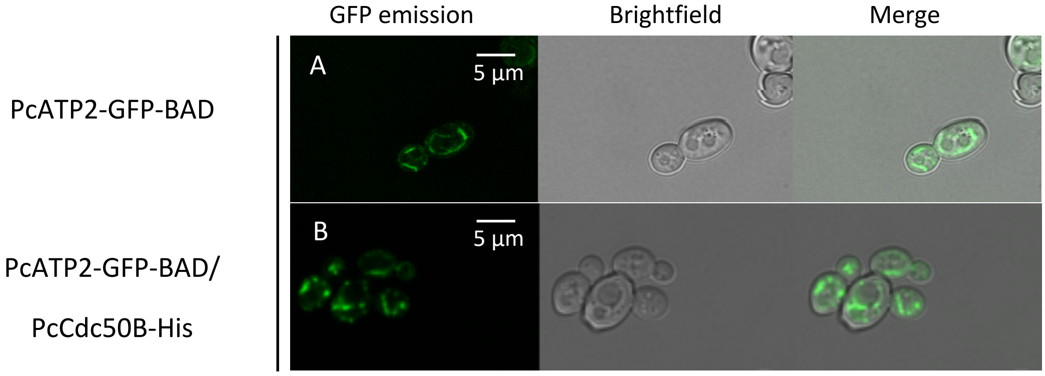

Localization in *S. cerevisiae* of GFP-tagged PcATP2 or PcCdc50B alone or co-expressed with their respective non-tagged partner.

Confocal GFP fluorescence microscopy images of *S. cerevisiae* cells expressing PcATP2-GFP-BAD (Panel A), and co-expressing PcATP2-GFP-BAD and PcCdc50B-His (Panel B). Pictures were taken with a Leica SP8 confocal microscope, using the 63x oil objective and an excitation wavelength of 488 nm.

Table supplement 1.

Detergents used in the membrane solubilization experiments.

	Mw (g/mol)[a]	CMC (mM)[b]
n-decyl-β-D-maltopyranoside (DM)	482.56	1.6
n-undecyl-β-D-maltopyranoside (UDM)	496.59	0.59
n-dodecyl-β-D-maltopyranoside (DDM)	510.62	0.17
5-cyclohexyl-1-pentyl-β-D-maltoside (CYMAL-5)	494.57	2.4
lauryl maltose neopentyl glycol (LMNG)	1000.19	0.01
n-Octyl-β-D-glucopyranoside (OG)	292.37	18
n-Octyl-β-D-thioglucopyranoside (OTG)	308.43	9
Laurydimethylaminoxide (LDAO)	229.4	1
n-dodecyl phosphocholine 12 (FosC12)	351.5	1.5
octaethylene glycol monododecyl Ether (C12E8)	538.75	0.09
3-[(3-Cholamidopropyl)dimethylammonio]-1-propanesulfonate hydrate (CHAPS)	614.88	8

[a, b] data extracted from the commercial suppliers listed in the methods section.

REFERENCES FROM SUPPLEMENTARY INFORMATION

1 Tsirigos KD, Peters C, Shu N, Käll L, Elofsson A. The TOPCONS web server for consensus prediction of membrane protein topology and signal peptides. *Nucleic Acids Res* 2015; **43**: W401-7.

2 Hiraizumi M, Yamashita K, Nishizawa T, Nureki O. Cryo-EM structures capture the transport cycle of the P4-ATPase flippase. *Science* 2019; **365**: 1149–1155.

3 García-Sánchez S, Sánchez-Cañete MP, Gamarro F, Castanys S. Functional role of evolutionarily highly conserved residues, N-glycosylation level and domains of the Leishmania miltefosine transporter-Cdc50 subunit. *Biochem J* 2014; **459**: 83–94.

4 Poulsen LR, López-Marqués RL, McDowell SC *et al.* The Arabidopsis P4-ATPase ALA3 localizes to the golgi and requires a β-subunit to function in lipid translocation and secretory vesicle formation. *Plant Cell* 2008; **20**: 658–676.

5 Timcenko M, Lyons JA, Januliene D *et al.* Structure and autoregulation of a P4-ATPase lipid flippase. *Nature* 2019; **571**: 366–370.
